# MaizeMine: A Data Mining Warehouse for the Maize Genetics and Genomics Database

**DOI:** 10.3389/fpls.2020.592730

**Published:** 2020-10-22

**Authors:** Md Shamimuzzaman, Jack M. Gardiner, Amy T. Walsh, Deborah A. Triant, Justin J. Le Tourneau, Aditi Tayal, Deepak R. Unni, Hung N. Nguyen, John L. Portwood, Ethalinda K. S. Cannon, Carson M. Andorf, Christine G. Elsik

**Affiliations:** ^1^Division of Animal Sciences, University of Missouri, Columbia, MO, United States; ^2^Division of Environmental Genomics and Systems Biology, Lawrence Berkeley National Laboratory, Berkeley, CA, United States; ^3^USDA-ARS Corn Insects and Crop Genetics Research Unit, Iowa State University, Ames, IA, United States; ^4^Division of Plant Sciences, University of Missouri, Columbia, MO, United States; ^5^MU Institute for Data Science and Informatics, University of Missouri, Columbia, MO, United States

**Keywords:** data mining, genome database, InterMine, maize, *Zea mays*

## Abstract

MaizeMine is the data mining resource of the Maize Genetics and Genome Database (MaizeGDB; http://maizemine.maizegdb.org). It enables researchers to create and export customized annotation datasets that can be merged with their own research data for use in downstream analyses. MaizeMine uses the InterMine data warehousing system to integrate genomic sequences and gene annotations from the *Zea mays* B73 RefGen_v3 and B73 RefGen_v4 genome assemblies, Gene Ontology annotations, single nucleotide polymorphisms, protein annotations, homologs, pathways, and precomputed gene expression levels based on RNA-seq data from the *Z. mays* B73 Gene Expression Atlas. MaizeMine also provides database cross references between genes of alternative gene sets from Gramene and NCBI RefSeq. MaizeMine includes several search tools, including a keyword search, built-in template queries with intuitive search menus, and a QueryBuilder tool for creating custom queries. The Genomic Regions search tool executes queries based on lists of genome coordinates, and supports both the B73 RefGen_v3 and B73 RefGen_v4 assemblies. The List tool allows you to upload identifiers to create custom lists, perform set operations such as unions and intersections, and execute template queries with lists. When used with gene identifiers, the List tool automatically provides gene set enrichment for Gene Ontology (GO) and pathways, with a choice of statistical parameters and background gene sets. With the ability to save query outputs as lists that can be input to new queries, MaizeMine provides limitless possibilities for data integration and meta-analysis.

## Introduction

Maize (*Zea mays* L. ssp. *mays)* is one of the most economically important grain crops in the world, serving as a source of food, feed, and fuel. Maize is also a leading model organism for studying a range of complex biological processes involving developmental physiology, genome evolution, epigenetics, heterosis, and plant domestication (Strable and Scanlon, 2009). The availability of the maize B73 genome sequence (Schnable et al., 2009) and numerous additional genomic resources has accelerated both maize breeding and genetics research. With the advent of single molecule sequencing technologies, the maize B73 reference genome has been improved with higher contiguity (Jiao et al., 2017). Multiple research groups have also put their efforts into developing improved versions of maize gene annotations (Law et al., 2015; Wang et al., 2016; Jiao et al., 2017). Genomics data generated by the maize research community are stored and curated in the USDA-ARS supported Maize Genetics and Genomics Database (MaizeGDB^1^) (Portwood et al., 2019).

With the ever-growing volume of available maize genomics data has arisen the need for an efficient means to integrate, access, and query the data. MaizeGDB has addressed these needs by establishing MaizeMine, a data mining resource based on the InterMine data warehousing system (Smith et al., 2012). The InterMine platform has been well received by the plant genomics community. Widely adopted plant-based InterMine databases include ThaleMine (Krishnakumar et al., 2017), and MedicMine (Krishnakumar et al., 2015), as well as SoyMine, CowpeaMine, PeanutMine, and BeanMine by the Legume Federation.^2^ In addition, PhytoMine, a multispecies InterMine, provides a cross-species comparative genomics interface (Goodstein et al., 2012).

Here, we present a data mining platform called MaizeMine^3^ for the maize genomics research community. It offers a broad range of functionality to handle a wide variety of genomic datasets. It provides options for analyzing lists, searching for genomic features within defined genomic regions, building complex queries, and performing enrichment analysis. Additionally, maize researchers can utilize their research data, or publicly available genomic datasets to perform meta-analyses using MaizeMine.

## Results and Discussion

### Data Sources

MaizeMine uses the InterMine data warehousing system to integrate genomic sequences and gene annotations from the B73 RefGen_v3 (Schnable et al., 2009) and B73 RefGen_v4 (Jiao et al., 2017) genome assemblies. The data sources are provided on the MaizeMine Data Source page, available using the Data Sources tab in the MaizeMine navigation bar (described below). MaizeMine includes three *Z. mays* gene sets: 5b+ (also called AGPv3) (Law et al., 2015), Zm00001d.2 (also called AGPv4) (Jiao et al., 2017), and NCBI RefSeq (Rajput et al., 2019), with database cross references connecting genes from equivalent loci. Data from external sources include Gene Ontology (GO) annotations from UniProt-GOA (Huntley et al., 2015; The Gene Ontology Consortium, 2019); protein annotations from UniProt (UniProt Consortium, 2019); protein families and domains from InterPro (Mitchell et al., 2019); single nucleotide polymorphisms (SNPs) from NCBI dbSNP (Sherry et al., 2001); pathways from CornCyc (Walsh et al., 2016), KEGG (Kanehisa et al., 2019), and Plant Reactome (Naithani et al., 2020); and publications from NCBI PubMed (Sayers et al., 2020). MaizeMine includes orthologs from Plant Ensembl Compara (Howe et al., 2020) for other monocot species and the model plant, *Arabidopsis thaliana*, enabling comparison across species. We have also incorporated several community datasets: Dissociation (Ds) transposable element insertion sites (Vollbrecht et al., 2010), Mutator (Mu) transposon insertion sites (Williams-Carrier et al., 2010), UniformMu transposon flanking sequence tags (Settles et al., 2007), ethyl methanesulfonate mutagenesis (EMS) mutation sites (Lu et al., 2018), Maize-GAMER GO annotations (Wimalanathan et al., 2018) and SNP alias ids from the Illumina 50K SNP array (Ganal et al., 2011). MaizeMine includes gene expression values for over 80 tissues computed for all three gene sets based on publicly available RNA-seq data (NCBI BioProject PRJNA171684) that had previously been generated for the *Z. mays* Gene Expression Atlas (Sekhon et al., 2013; Stelpflug et al., 2016). Along with the gene expression data, MaizeMine includes associated metadata from NCBI Sequence Read Archive (SRA) and BioSamples database, as well as Plant Ontology (Cooper et al., 2013) terms curated based on information from the SRA and the *Z. mays* Gene Expression Atlas publications (Sekhon et al., 2013; Stelpflug et al., 2016).

### Home Page, Navigation, and Quick Search

**FIGURE 1:**
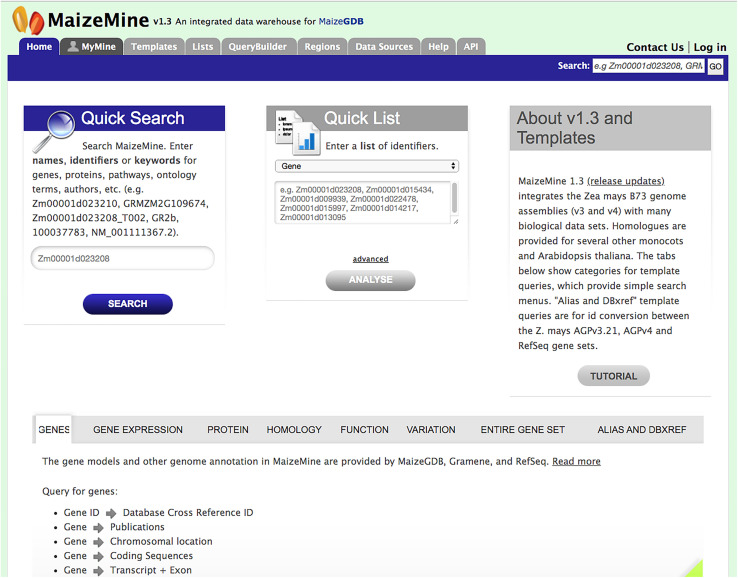
The MaizeMine home page allows users to initiate analysis by using a query form for “Quick Search” or “Quick List” or by using predefined template queries organized into major data categories in the lower portion of the homepage. The MaizeMine navigation bar at the top of the page provides access to the more advanced search options, QueryBuilder, List Tool, and Regions search tools.

MaizeMine is accessible from the MaizeGDB homepage via a quick link button. The MaizeMine navigation bar is available at the top of every MaizeMine page, with tabs for various search tools, the Data Source page, the Help page, the MyMine user account and the application programming interface (API) (Figure 1). The Help page provides detailed examples of all the MaizeMine tools and is a good place to start for an overview. The Quick Search tool on the MaizeMine home page (Figure 1 and Supplementary Figure 1) also provides a way to become acquainted with the database contents before performing more complex queries. It performs a full text search of all MaizeMine datasets and supports wildcards. The result of a Quick Search is a list of entities containing the searched term, to the left of which is a tool for filtering by data category or organism (Supplementary Figure 1). The Quick List tool on the MaizeMine home page (Figure 1) accepts lists of gene or protein identifiers, and is a slimmed down version of the List Tool, which is described below. In addition to the Quick Search and Quick List tools, midway down the MaizeMine home page tabs are provided to organize predefined template queries (described below) into categories (GENES, GENE EXPRESSION, PROTEIN, HOMOLOGY, FUNCTION, VARIATION, ENTIRE GENE SET, and ALIAS AND DBXREF) (Figure 1). The ENTIRE GENE SET category contains template queries similar to those in other categories, but constrain the searches to an entire gene set rather than specified gene identifiers.

### Report Page

MaizeMine provides a report for each entity in the database that can be accessed either by performing a Quick Search or by clicking an identifier in any query output. As an example, the Gene Report Page (Supplementary Figures 2–4) is divided into multiple sections: “Summary,” “Gene,” “Gene Expression,” “Protein,” “Function,” “Homology,” “Publications,” presented as a collection of tables, which can be downloaded in various formats. The Summary section provides gene identifiers, symbols, description, organism, chromosome, strand, and other identifiers such as aliases and database cross references (Supplementary Figure 2). The Transcripts section provides information about the gene model (transcripts, exons, coding sequences) and gives a visual representation of the gene model highlighting the structure of the gene (Supplementary Figure 2). You can download FASTA-formatted sequences for each type of feature provided in the gene model section. The Gene Expression section provides normalized read counts and FPKM values for RNA-seq data along with tissue description, growth stage and Plant Ontology terms (Supplementary Figure 3). The Protein section includes protein name, accession and length (Supplementary Figure 3). The protein ids and accession numbers are linked to Protein Reports, with more information including protein features and domains, and keywords and comments from UniProt. The Function section (Supplementary Figure 3) provides pathways and GO annotation with evidence codes. The Homolog section lists plant homologs with the source of homolog information (Supplementary Figure 4). The Publication section provides publications from PubMed (Supplementary Figure 4).

### MyMine

MaizeMine can be explored anonymously, but a MyMine account is required for saving lists, queries and query templates for later use. Clicking “Log In” on the upper right of the home page provides access to a link for creating a new account, which requires simply an email and password. Once an account is created, the MyMine tab in the navigation bar can be used to access user-specific information, including lists, a query history for the current session, saved queries and saved templates. The Account Details tab allows you to change your public username for sharing lists with other users. It also provides options to generate an API key for programmatic access and to enter a URL to enable sending query outputs to a Galaxy instance (Jalili et al., 2020).

### Template Queries

**FIGURE 2:**
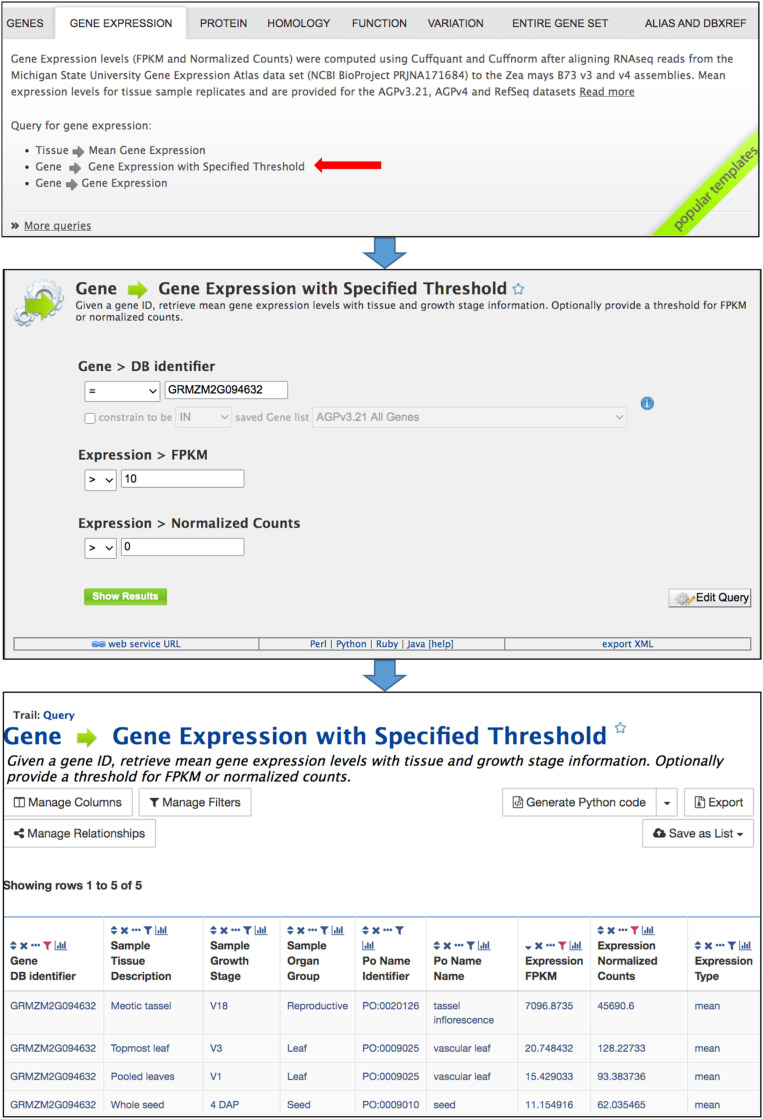
Template queries are most easily accessed by selecting the appropriate template category tab in the middle of the home page. Clicking a template query name opens a simple menu where constraints are entered. The query is performed after clicking “Show results.” The menu also provides an “Edit Query” button which enables modification of the query using the QueryBuilder, as well as access to client library code for the API by clicking “Perl,” “Python,” “Ruby,” or “Java” in the bar near the bottom. The query output is a table that can be modified by sorting and filtering using icons in the column headers. The Manage Columns, Manage Filters, and Manage Relationships buttons provide additional table manipulations, such as adding columns. The table can be exported using the Export button.

Predefined template queries, available either using the Template tab in the main navigation bar or by selecting a template category tab in the middle of the MaizeMine home page, provide an easy means of performing complex queries (Figure 2). Clicking a template query name provides a simple interface that is pre-populated with one or more example constraints, and may include pull-down menus (Figure 2). You can perform the query using the default identifier or enter a different value. If you are logged in and have already saved a list (described below), an additional option will allow the input of the list to constrain multiple searches. Additional constraint options may include numerical operations, such as “less than,” “greater than,” and “not equal to.” Results are obtained by clicking the “Show Results” button. Alternatively, clicking “Edit Query” provides access to the QueryBuilder for query modification (described below), which allows removal or addition of search constraints and output attributes. The query output is in a tabular format that can be manipulated by filtering, sorting, and column reordering, and can be downloaded in various formats, including tab delimited, GFF3, BED, XML, and JSON.

### QueryBuilder

**FIGURE 3:**
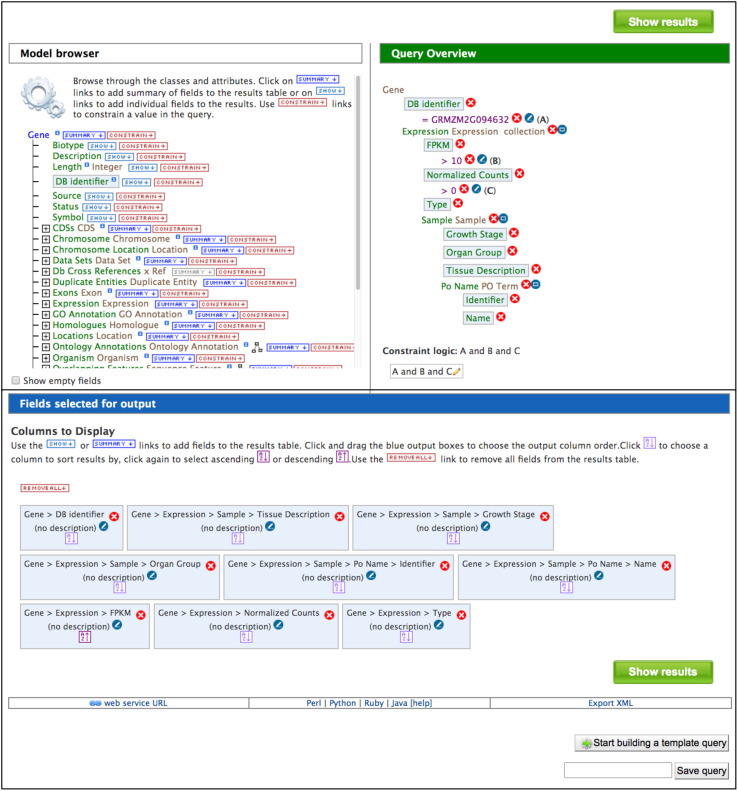
The Model Browser and Query Overview are part of the QueryBuilder, which allows users to develop custom queries. Clicking “CONSTRAIN” and “SHOW” in the Model Brower adds constraints and output fields, respectively, to the query shown in the Query Overview. Light blue boxes in the Model Browser and Query Overview indicate attributes that have been selected for output. The output columns and their order in the final results table are illustrated by blue boxes in the “Fields selected for output” section. In addition to running the query, a custom query can be named and saved, exported for sharing and developed into a template query. Below the “Fields selected for output” section is a bar that provides access to client library code for the API by clicking “Perl,” “Python,” “Ruby,” or “Java”.

The QueryBuilder is used to modify template queries or construct custom queries. The QueryBuilder does not require previous database programming experience, but it does require practice. Modifying template queries is a good way to become familiar with the QueryBuilder prior to constructing a query from scratch. To modify an existing template query, click the “Edit Query” button in a template menu (Figure 2). This leads to a page with the Model Browser, Query Overview and a display of fields selected for output (Figure 3). The Model Browser shows a hierarchical data model rooted at the data class that is the main subject of the query. Each class can be expanded to show attributes and subclasses. Clicking either the word “constrain” or “show” next to an attribute adds constraints and output fields, respectively, to the query shown in the Query Overview. Selecting “constrain” causes a menu to appear that allows entry of a constraint identifier or selection of a constraint from a pull-down menu. Clicking on “show” next to a class attribute adds the attribute as an output column for the query, shown in a blue box in the Query Overview. A constraint or output field can be removed from the query by clicking the associated red X in the Query Overview. A constraint can be edited by clicking the associated pencil symbol in the Query Overview. Once query modification is complete, the “fields selected for output” section below the Model Browser shows boxes that signify output columns and can be dragged to rearrange column order. Alternatively, boxes can be eliminated from the output by clicking on the red X just to the right of the box. You can download the query as XML to share with others, and if you are logged in, you can save the query and create a template for the query.

To construct a *de novo* query rather than altering an existing template query, use the QueryBuilder tab in the navigation bar for direct access to the QueryBuilder page (Supplementary Figure 5), where links are available for browsing the data, importing a query, initializing a new query, and viewing your query history. Clicking “Browse the Data Model” allows you to navigate the hierarchical structure of data objects (classes) and sub-classes (Supplementary Figure 6) of the entire database. The largest class in MaizeMine is “Bio-Entity,” which includes the classes “Protein,” “Protein Domain,” and “Sequence Feature.” The “Sequence Feature” class is further divided into classes, such as “Gene” and “Transcript.” Clicking a class in this tree opens the Model Browser (described above). Rather than searching through the entire data hierarchy, a simpler way to access the Model Browser from the QueryBuilder page is to select a class in the pull-down menu on the right side of the page (Supplementary Figure 5). Upon doing so, you will notice that the Query Overview is blank. Initiate query construction by clicking either the word “show” or the word “constrain” next to a class, similar to the approach used in modifying a template query (described above).

### List Tool

**FIGURE 4:**
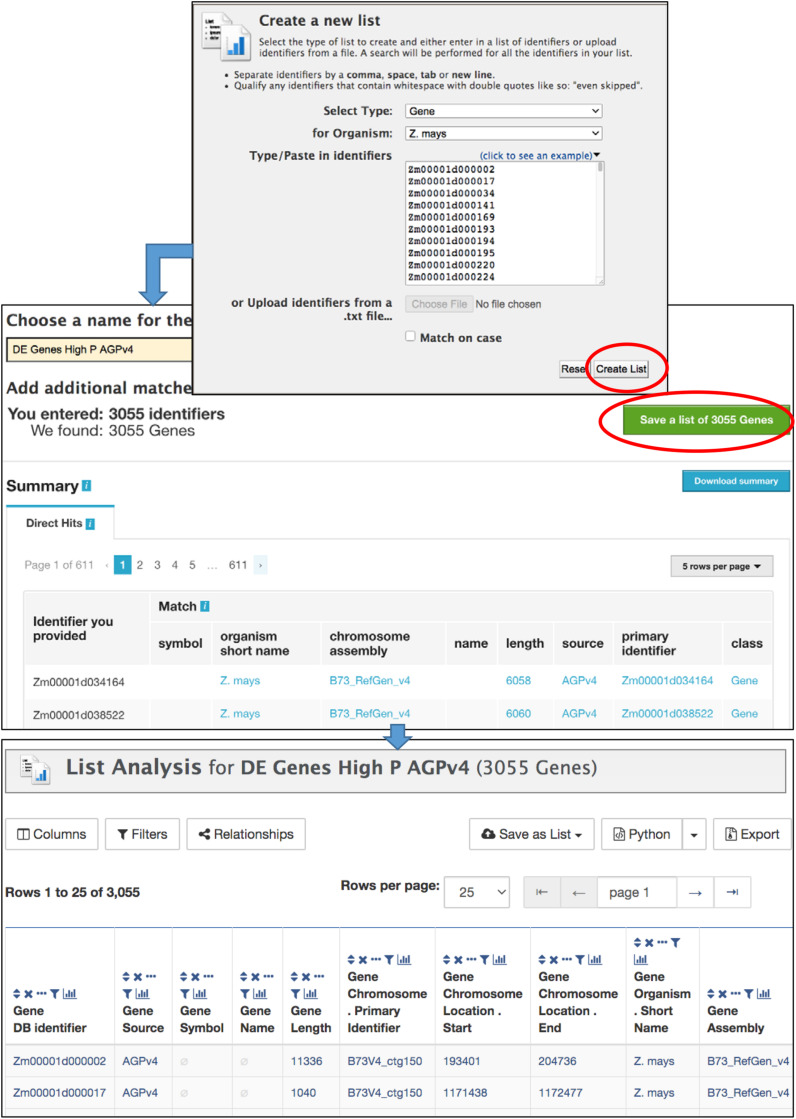
The List Upload menu is part of the List Tool, and allows the upload of identifiers for any MaizeMine data type. After the list is submitted using “Create List,” the database verifies the presence of the identifiers prior to prompting the user to enter a name for the list, resolve duplicated identifiers if necessary, and save the final list. Upon saving, the List Analysis page appears, with a table showing default information about the list. If the list comprises of genes, gene set enrichment widgets appear below the table (not shown).

The List Tool allows you to create and manage lists of identifiers that can be used in further analysis (Figure 4). It is important to be logged in to your MyMine account (described below); otherwise your lists cannot be saved beyond the current session. Once a list is saved, it can be used in list operations and queries, can be shared with other users, and remains available in future sessions. The List Tool can be accessed using the Lists tab in the navigation bar or by clicking “advanced” in the Quick List box on the home page. The List Tool opens to either a List Upload page or a List View page. A blue bar below the MaizeMine navigation bar provides the links “Upload” and “View” to toggle between the pages. On the List Upload menu, the Select Type pull-down menu allows you to set the data type for the identifiers to be entered. Selecting an organism is not necessary if the identifiers are unique, but will speed up the search. You can either enter a list of identifiers into the text box in a single column or separated by commas, or upload a list of identifiers as a text file. After clicking “Create List,” the database performs a lookup to verify the presence of the identifiers. The number of identifiers entered and found are each reported (Figure 4), and missing identifiers are listed at the bottom of the page. You are prompted to disambiguate any identifiers found in multiple MaizeMine datasets, and can select which to include in your final list. The ability to disambiguate duplicate identifiers is important in MaizeMine, because some gene ids and gene symbols are found in multiple maize gene sets. Before saving the list by clicking “Save a list of…Genes,” you should enter a concise and unambiguous name for the list so it can be easily recognized in later analyses. Once the list has been saved, the List Analysis page with an output table showing default information specific to the data type appears (Figure 4), but may take a few moments depending on the number if identifiers entered. A message at the top of the page will indicate the list has been saved. Clicking that message or clicking the View toggle link will lead to the List View page. Below the table is a text entry box to provide a description for the list. The Tags menu allows the creation of new descriptive tags and addition of tags to the list, to enable tag-based list filtering on the List View page. Scrolling down the List Analysis page will reveal gene set enrichment widgets, available only for gene lists (described below).

**FIGURE 5:**
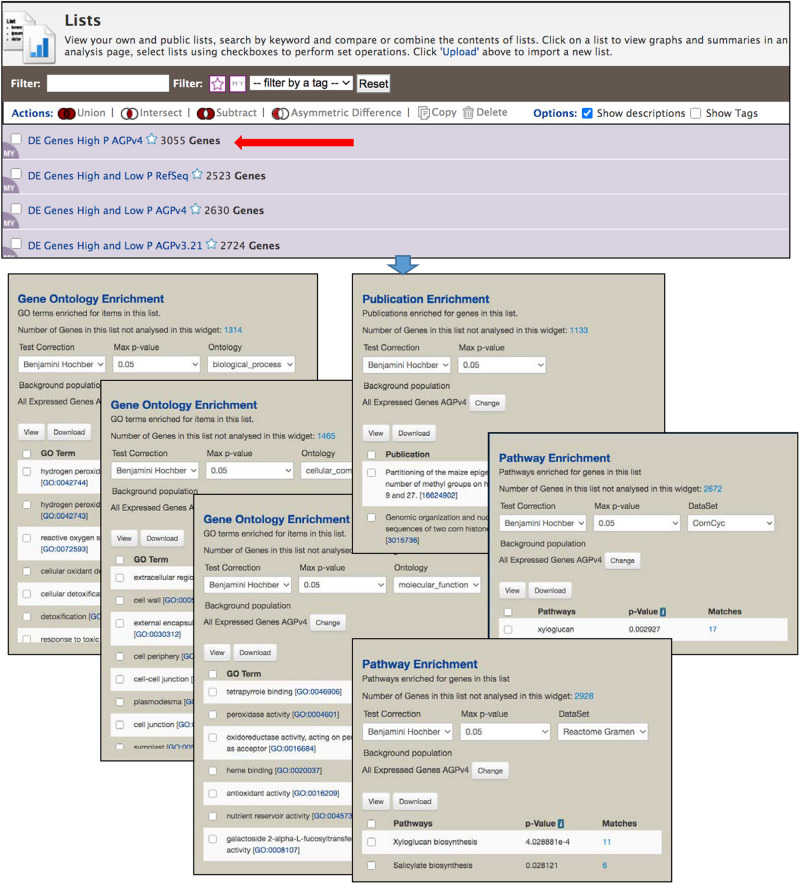
The List View page is part of the List Tool, and allows user to view the saved lists, perform set operations among lists and delete lists. Clicking the name of a list opens the List Analysis page, at the top of which is the table shown in Figure 4. Here, the gene set enrichment widgets below the table in Figure 4 are shown. Enrichment is automatically performed each time a gene list is selected, using an InterMine default background population based on all genes for an organism. However, in MaizeMine it is essential that a more appropriate background population be selected using the “Change” button, because MaizeMine includes three *Z. mays* gene sets. This figure shows enrichment for all three GO ontologies (selected under “Ontology”), both Reactome and CornCyc pathways (selected under “DataSet”), and PubMed publications. Results of each enrichment can be downloaded in tab delimited format using the “Download” button.

The List View page (Figure 5) shows the saved user lists, with a mauve background, as well as default gene set lists available to all users, with a white background. Clicking an individual list returns to the List Analysis page. Stars next to list names allow you to indicate favorite lists. At the top of the List View page are actions, including set operations, that can be performed on selected lists. Lists can be filtered based on tags and favorite status.

### Enrichment Widgets

The List Analysis page provides gene set enrichment widgets for lists of gene identifiers, shown below the list output table. Widgets are provided for GO, pathway and publication enrichment (Figure 5). Each widget includes pull-down menus to select the test correction and maximum *p*-value. The GO and pathway widgets also include a pull-down menu for the specific ontology or the pathway data source, respectively. Different pathway datasets are available for various gene sources, as shown on the Data Source page (in the “Organism” column). CornCyc and Reactome are available only for AGPv4; KEGG is only available RefSeq. AGPv3.21 genes are not connected to any pathway dataset, so will always result in no pathway enrichment. The background population for enrichment can be changed using the “Change” button, which provides access to a pull-down menu that allows selection of a user-saved gene list or an entire gene set. By default, InterMine uses all annotations (e.g., all GO terms) for a particular organism as the background for an enrichment test. Since MaizeMine includes multiple maize gene sets, the InterMine default is not the appropriate background and may result in false positives. Therefore, you should always change the background gene list to either a list you have already saved or one of the individual gene sets. The background gene list must be changed for each widget individually. *p*-values for enrichments are computed using a hypergeometric distribution and the selected test correction. Enrichment results can be downloaded as a tab-delimited text file by clicking the Download button.

### Regions Search

**FIGURE 6:**
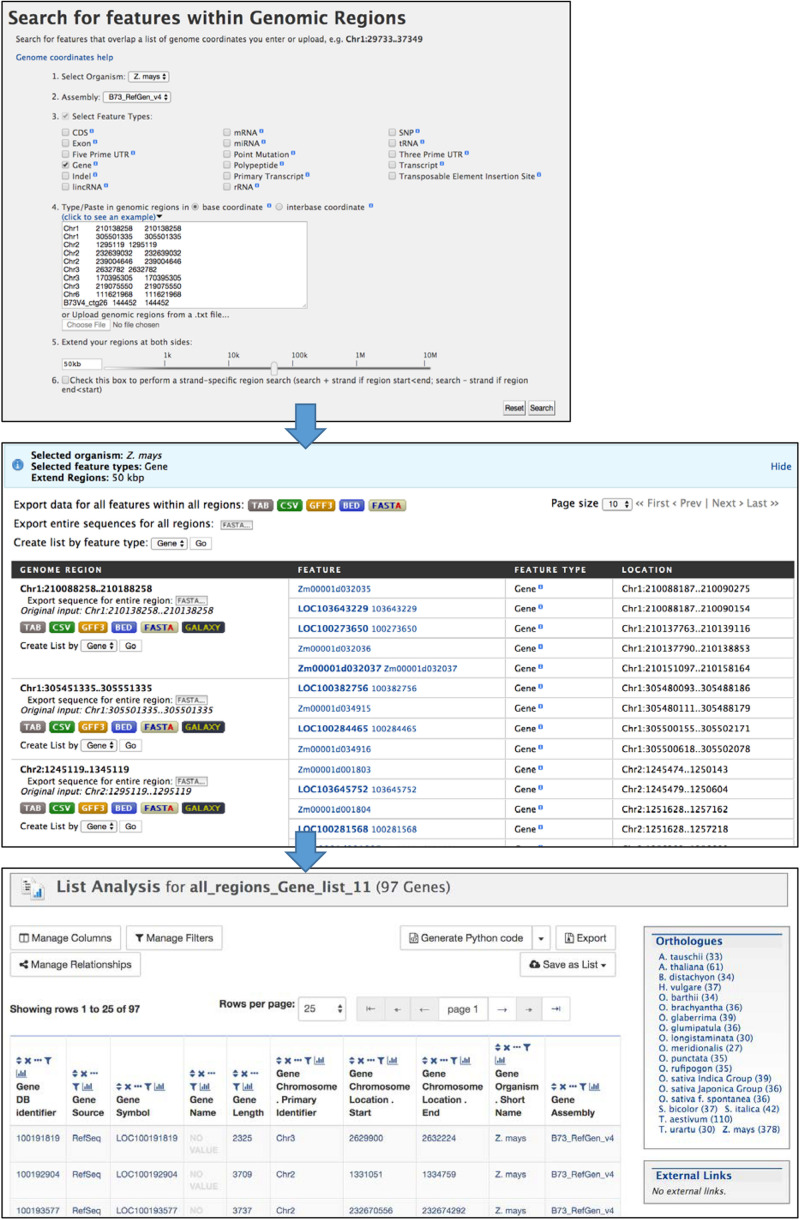
The Regions Search menu takes chromosome coordinate input lists in several formats to retrieve selected features within those regions of the specified genome assembly. The input format requires both a start and end coordinate, so in the case of SNPs the same coordinate is entered twice. The regions can be extended using the entry box or slider bar near the bottom of the menu. The output of the search consists of pages showing results for each region. Results can be downloaded in various formats for each region separately or altogether. The “Create List by feature type” function allows you to create a list of identifiers, and leads to the List Analysis page.

The Genomic Region search tool (Figure 6) accessed through the “Regions” tab allows you to perform a location-based search (chromosome and coordinates) for genomic features. For example, you can search for genes within a specified distance of significant GWAS SNPs. The maize assembly version is selected with a pull-down menu, then features for output are selected using checkboxes and search coordinates are entered in the text box or by uploading a text file. The genomic interval can be extended by entering a distance (e.g., 50 kb) into the box or by using the slider bar. The search is not strand-specific by default, but an option is provided to make it strand-specific. The output of the Regions search is a page showing selected features for each region, with options to download results separately by region or altogether in various formats. The Regions output page provides the option to create a list, which automatically opens the List Analysis page for further analysis in MaizeMine.

### Application Programming Interface

The InterMine platform provides a web service API that enables users to automate workflows and access data without using the webapp. The InterMine web services provide all functionality that is available in the webapp, with client library support in Python, Perl, Java, JavaScript, Ruby, and R (Kalderimis et al., 2014; Kyritsis et al., 2019).^4^ Prior to using the MaizeMine API, you should login to MaizeMine and generate an API key on the “Account Details” page under the MyMine tab. The generated API token can be used in place of user credentials in API scripts. The API tab in the MaizeMine navigation bar provides access to pages describing how to get started with the Perl, Python, Java, and Ruby client API libraries. To become familiar with the API, you can retrieve an automatically generated script to run a query by clicking “Perl,” “Python,” “Ruby,” or “Java” in the bar near the bottom of any template query menu (shown in Figure 2) or below the “Fields selected for output” section in the QueryBuilder interface (shown in Figure 3). MaizeMine API examples are provided in Supplementary Data Sheet 2.

### MaizeMine Use Examples

We provide example use cases that demonstrate how MaizeMine can be used with researcher-supplied datasets. These examples were developed using MaizeMine v1.3; results may vary when using future MaizeMine releases. Performing these examples while logged in to a MyMine account will allow you to continue your work in future sessions should you be interrupted. The first example demonstrates the Regions Search Tool. The second example demonstrates the List Tool and the “Gene ID → Database Cross Reference ID” template query. Both examples make use of the Gene Set Enrichment Widgets.

#### Example 1: Regions Search

This example demonstrates the use of the Regions Search Tool to identify and save a list of genes located within 50 kb of SNPs found to be associated with the trait “water content of shoots and leaves” (Yan et al., 2017) and how to perform GO and pathway enrichment analyses. The steps for this example are illustrated in Figure 6 and Supplementary Figures 7–10. Because the original study was done using the B73 RefGen_v2 assembly, we downloaded the SNP locations from the GWAS Atlas,^5^ which had been curated and mapped to B73 RefGen_v4 (Tian et al., 2020). The mapped locations are provided in Supplementary Table 1.

1. After logging in to MaizeMine, click the Regions tab in the main navigation bar at the top of the page. In the Regions search menu (Figure 6), *Z. ma*ys is already selected as the organism. Next to “2. Select Assembly,” use the pull-down menu to select “B73_RefGen_v4.” Click the box next to “3. Select Feature Type” to unselect all the features; then check the box next to Gene. Paste the SNP locations from Supplementary Table 1 into the text box under “4. Type/paste in genomic regions” and leave “base coordinates” selected. One of the accepted formats for chromosome locations is chromosome id, start coordinate and end coordinate separated by tabs. Note that for a SNP location, both start and end coordinates are required even though they are identical. To extend the search region from the entered coordinates, enter 50 kb in the box below “5. Extend your regions at both sides.” Alternatively, you can use the slider bar to extend the regions. The last box, number 6, allows you to perform a strand-specific search. For this example, the box is left unchecked. Finally, click Search to perform the search.
  The search may take several minutes depending on the size of the regions searched, with progress shown in a bar near the top of the page. The completed search result is a list of genes found in each region; genes can be downloaded for individual regions in various formats, or downloaded for the entire search using buttons at the top of the page. For further MaizeMine analysis, a list of genes is created by clicking “GO” next to “Create list by feature type,” bringing up the List Analysis page. Rather than having the option to name the list, the list is automatically named “all_regions_Gene_list” with a consecutive unique digit, depending on how many similar lists you already have saved. You can edit the name later under the Lists tab in your MyMine account.
2. On the List Analysis page, the Gene list output includes the gene source, which is necessary for further analysis of the Regions Search results, because the Regions Search for genes is not limited to a particular gene set. Clicking the histogram icon above the Gene Source column shows that there are 53 and 44 genes from the AGPv4 and RefSeq gene sets, respectively (Supplementary Figure 7). Scrolling down past the table shows the enrichment widgets, which always appear when a gene list is saved. It is important to ignore the enrichment widgets when the list contains genes from multiple gene sets, because combining gene sets causes incorrect GO term counts and may result in false positive enrichment. Instead, new lists are saved for each gene set separately. To do so, click the histogram icon above the Gene Source column and check “AGPv4,” click the arrow next to “Filter” and then select “Restrict Table to Matching Rows.” Once the table has been restricted to only AGPv4 genes, save the list by clicking “Save as List” above the table, and then click “Gene (53 Genes).” A window pops up allowing you to enter a name for the list. Name it “Genes within 50 kb water content of shoots and leaves AGPv4.” After clicking “Create List,” confirmation that a new list has been saved will appear below the navigation bar. In order to go back and also save the RefSeq gene list, click the Undo button to undo the previous filtering step. Use the histogram icon above the Gene Source column once again to filter the list, but this time select RefSeq genes. Once the table has been filtered, use the “Save as List” button again to save 44 RefSeq genes using the name “Genes within 50 kb water content of shoots and leaves RefSeq.” After both lists have been saved, you can view all of your saved lists by clicking “View” under the navigation bar.
3. Enrichment analysis can be performed once the gene lists have been saved for individual gene sets. Clicking the list “Genes within 50 kb water content of leaves and roots AGPv4” brings back the List Analysis page, with a new gene-set specific enrichment analysis (Supplementary Figure 8). Scroll down to the enrichment widgets (Supplementary Figure 9).
  Gene Ontology enrichment may take a few moments, so go down to Pathway enrichment. When performing gene set enrichment in MaizeMine, it is essential that the background population be changed from the default, which includes all genes from all three maize gene sets, to a more appropriate population. For a Regions Search, it would be appropriate to use all the AGPv4 genes in the genome. After clicking “Change” next to “Default” a pull-down menu will allow you to select “AGPv4 All Genes,” which is a default gene list present in all user accounts. The enrichment will be recomputed with the selected background gene list. Pull-down menus also allow you to change the test correction and maximum *p*-value. For pathway enrichment, MaizeMine provides a choice of three pathway datasets, but each is available for only one of the three gene sets. KEGG is the default pathway setting, but is available only for RefSeq genes. Therefore, the “Number of Genes in this list not analyzed in this widget” is 53, the total number of genes in the list. Both CornCyc and Reactome are available for a small subset of genes in the AGPv4 annotation, as indicated by the “Number of Genes in this list not analyzed by this widget” (51 and 50 for CornCyc and Reactome, respectively). Use the pull-down menu to change the pathway dataset. In this example, we find enrichment for a Reactome pathway, “Cyanate degradation,” but no enrichment for CornCyc pathways. It is not unusual for datasets with small gene numbers to show little or no enrichment. The Reactome enrichment result can be downloaded as a text file using the download button.
  Go back to the GO enrichment widget. The background population again needs to be changed to “AGPv4 All Genes.” With the default test correction (Holm–Bonferroni), the enrichment for Biological Process GO terms shows enrichment for four terms – “response to carbon dioxide,” “regulation of stomatal movement,” “stomatal movement,” and “carbon utilization.” When using the slightly less stringent Benjamini Hochberg test correction, “response to stimulus” also appears as an enriched term. Using the pull-down menu to change the ontology shows that there are no enriched terms for Cellular Component, but five enriched terms for Molecular Function when the Benjamini Hochberg test correction is still selected – “hydro-lyase activity,” “carbonate dehydratase activity,” “carbon-oxygen lyase activity,” “protein deglycase activity,” and “lyase activity.”
  The Publication enrichment widget does not show enrichment with either default settings or after changing the background population to “AGPv4 All Genes.” Changing the test correction to “None” shows three publications, but the generally accepted approach in gene set enrichment analysis is to use a multiple test correction to avoid false positives. Similarly, publications appear in the list after increasing the maximum *p*-value to 1, but keep in mind that high *p*-values are not significant, and 0.05 is a commonly accepted *p*-value cutoff.
4. Once the enrichment results for AGPv4 have been downloaded by clicking Download, you can go back to the List View page, click on the “Genes within 50 kb water content of shoots and leaves RefSeq” and perform enrichment analysis for the RefSeq genes using methods described in step 3. In this case, you will use “RefSeq All Genes” as the background gene list in each enrichment widget (Supplementary Figure 10). You will find the gene list is enriched for the KEGG term “Nitrogen Metabolism.” The CornCyc and Reactome datasets are not available for RefSeq genes. For GO enrichment using the Benjamini Hochberg test correction, you will find two enriched terms for Biological Process – “carbon utilization” and “response to carbon dioxide,” and the same 5 enriched Molecular Function terms found with the AGPv4 list. It is not surprising that the GO enrichment results are slightly different between AGPv4 and RefSeq. Both the gene models and the sources of GO annotation differ between the two gene sets.

#### Example 2: List Tool

This example demonstrates use of the List Tool for enrichment analysis of a dataset of genes differentially expressed between maize axial and lateral roots under both low and high soil phosphorus conditions using published data available in a supplementary spreadsheet of a freely accessible article (Yu et al., 2018). It also shows the use of a template query to retrieve database cross references for gene ids in alternative maize gene sets. Unlike the Regions Search example above, in which genes from two MaizeMine gene sets are identified in the initial search, this example starts with a list of identifiers from one gene set (AGPv3.21) and uses a template query to retrieve cross reference ids for the two newer gene sets (AGPv4 and RefSeq) prior to further analysis. Download the Supplementary File, nph14893-sup-0002-notess1-s5.xlsx^6^ (Yu et al., 2018). The steps in this example are illustrated in Supplementary Figures 11–19 and Supplementary Table 2 provides results of the enrichment analyses.

1. The first step is to create a gene list that will serve as the background gene list for enrichment analysis. In this case, we will use all expressed genes from the previously published Supplementary Excel File described above (Notes S3 tab) (Yu et al., 2018). Click the List Tab in the MaizeMine navigation bar. If necessary, click “Upload” in the blue bar below the navigation menu to toggle to the List Upload menu. Choose “Gene” from the “Select Type” pull-down menu and “*Z. mays*” from the “Organism” menu (Supplementary Figure 11). Select all Gene IDs from column B of the Notes S3 worksheet of the Supplementary Excel File and paste them into the List Tool text box. Click “Create List.” Once the database lookup has completed, which may take a few moments, enter a name for the list: “All Expressed Genes AGPv3.21.” Notice that duplicates of the id “GRMZM2G467671” were found, because it is both a primary id in the AGPv3.21 gene set and a gene symbol in the RefSeq gene set. Click “Add” in the row for the AGPv3.21 gene so that it will be included in your final saved list (Supplementary Figure 11). Scrolling further down this page shows some identifiers that were not found in the database (Supplementary Figure 11). Click “Save a List of 27356 Genes” (Supplementary Figure 12). Once the list has been saved, you can click “View” in the blue bar below the navigation bar to view your lists. The new list appears at the top.
2. Convert AGPv3.21 gene identifiers to AGPv4 identifiers using a template query as follows. On the MaizeMine home page, select the ALIAS AND DBXREF template category tab, and then click the “Gene ID → Database Cross Reference ID” template query. Under “Gene > DB identifier,” click the box next to “constrain to be” and then select the list “All Expressed Genes AGPv3.21” that you created in the previous step (Supplementary Figure 13). Turn on the optional “Gene > Source” constraint by clicking “On” under the word “optional.” Leave the default values “=” and “AGPv3.21.” We are constraining this search to only genes in AGPv3.21, because some gene ids are used in multiple gene sets and would cause extra output rows. Click “Show Results.” Above the output table, select “Save as List,” and then highlight “Gene > DB Cross References > Cross References (25,022 Genes)” before clicking “Create List.” Enter a new name for the list: “All Expressed Genes AGPv4” (Supplementary Figure 14).
3. Similar to step 2, convert AGPv4 identifiers to RefSeq identifiers using the “Gene ID → Database Cross Reference ID” template query. This time the query is constrained to “All Expressed Genes AGPv4” and the optional Gene Source constraint is set to “AGPv4.” In the output table, the “Cross Reference Source” column shows two different gene sets in the result, because in MaizeMine AGPv4 gene ids are connected to both AGPv3.21 and RefSeq ids. The histogram icon above the “Cross Reference Source” column is used to filter for only RefSeq ids. Click the histogram icon, then check the box next to “RefSeq,” and click Filter (Supplementary Figure 15). With only RefSeq ids in the table, save the 23,624 Cross Reference Gene ids similar to step 4, naming the new list “All Expressed Genes RefSeq.”
4. Now that the gene lists (AGPv3.21, AGPv4, and RefSeq) to be used as the background for enrichment analyses have been saved for all three gene sources, it is time to upload the list of differentially expressed genes. The differentially expressed gene list we will use is Column L (Overlapping genes among root types under both P conditions) of the Notes S5 worksheet in the Supplementary Excel File downloaded at the start of the example. Go back to the List Upload page by clicking Lists in the navigation bar and toggling to “Upload” in the blue bar below the navigation bar, if necessary. Use methods similar to step 1 to upload the gene ids and save the list as “DE Genes High and Low P AGPv3.21.” In this case, 2724 gene ids are uploaded and there is one duplicate id that needs to be added by clicking “Add” in the AGPv3.21 row prior to saving the list of genes.
5. Repeat steps 2 and 3 to create new differentially expressed gene lists for the AGPv4 and RefSeq gene sources starting with the gene list “DE Genes High and Low P AGPv3.21” as input when you run the “Gene ID → Database Cross Reference ID” template query the first time. Name the new lists “DE Genes High and Low P AGPv4” and “DE Genes High and Low P RefSeq,” respectively.
6. Now that the gene lists for the alternative gene sources have been saved, enrichment analyses can be performed for each of the three sources by clicking on the name of each list in the List View page (Supplementary Figure 16). At this point you will notice that the number of differentially expressed genes differs among the three gene sources, due to differences in the predicted gene sets. The advantage of using different gene sources is that different sources are connected to different pathway datasets or publications, and the number of genes annotated with GO differs among gene sources. Click the name of the “DE Genes High and Low P AGPv3.21” gene list to go to the List Analysis page and view the enrichment widgets below the table. For the GO enrichment widget, change the background population to “All Expressed Genes AGPv3.21.” Notice that changing the background population has a noticeable effect on the list of GO Biological Process terms, illustrating the importance of selecting an appropriate background gene list. Using the Benjamini Hochberg test correction, the enriched Biological Process terms are “oxidation-reduction process” and “response to wounding” (Supplementary Figure 17). There are six enriched Cellular Component terms and 27 enriched Molecular Function terms (Supplementary Table 2). Pathway enrichment cannot be performed for this gene list, because MaizeMine does not include pathway information for AGPv3.21.
7. Go back to the List View page by clicking “View” in the blue bar below the navigation bar. Perform the gene enrichment analysis for the “DE Genes High and Low P AGPv4” using methods similar to those in step 6. Using the “All Expressed Genes AGPv4” background population and the Benjamini Hochberg test correction, there are many more significant Biological Process terms (46) than found in AGPv3.21 (Supplementary Figure 18 and Supplementary Table 2). Similarly, there are more GO Cellular Component (12) and Molecular Function (56) terms for AGPv4 compared to AGPv3.21 (Supplementary Table 2). The increase in significant GO terms is likely due to the higher proportion of AGPv4 genes annotated with GO terms compared to AGPv3.21. The AGPv4 gene list is also enriched for six CornCyc pathways and one Reactome pathway (Supplementary Figure 18).
8. Repeat step 7 for the “DE Genes High and Low P RefSeq” gene list, making sure to set the background population to “All Expressed Genes RefSeq.” The numbers of enriched GO terms is similar to those for the AGPv4 gene list, with 48 Biological Process, 8 Cellular Component, and 41 Molecular Function terms (Supplementary Figure 19 and Supplementary Table 2). The RefSeq gene list is also enriched for 13 KEGG pathways (Supplementary Table 2).

## Concluding Remarks

We have developed MaizeMine, a genomic data mining warehouse for *Z. mays* that enables researchers without programming skills to integrate their datasets with genome annotation data from a variety of external sources. MaizeMine currently supports two *Z. mays* B73 genome assemblies (B73_RefGen_v3 and B73_RefGen_v4) and three gene sets (AGPv3.21, AGPv4, and RefSeq). The transition to a new genome assembly can be difficult for researchers in the midst of ongoing projects, but MaizeMine can ease the transition by providing template queries that support conversion between gene sets. The use examples show it is advantageous to investigate all available gene sources, because different gene sources are associated with different ancillary datasets, such as CornCyc or KEGG pathways. We have also shown how genes from a supplemental dataset published using an older gene set can be reanalyzed using new gene annotations. The ability to save lists of identifiers, either by uploading ids or saving ids from query results and Regions searches, combined with the potential to perform bulk complex queries using entire lists, provides powerful means to integrate novel research data with a variety of genome annotation sources and published community datasets.

We will continue to enhance MaizeMine by incorporating new genome assemblies and datasets as they become available, and will continue to make source code modifications for MaizeMine available through GitHub.^7^ MaizeMine will have an annual release cycle. Future releases will fully support the then-current version of the maize reference genome, and will also make available (up to 5 years) at least one previous version of the reference genome. We look forward to receiving feedback from the maize research community and dataset suggestions for future MaizeMine releases.

## Materials and Methods

### Data Collection

*Zea mays* genome assemblies (B73_RefGen_v3 and B73_RefGen_v4) and their associated gene sets (called AGPv3.21 and AGPv4, respectively, in MaizeMine) were downloaded from the MaizeGDB.^8^ The gene sets called AGPv3.21 and AGPv4 in MaizeMine are equivalent to the 5b+ and Zm00001d.2 annotations, respectively, at the MaizeGDB. The RefSeq gene set was downloaded from NCBI.^9^ Datasets from other external sources were downloaded from their ftp or websites, as listed on the MaizeMine Data Source page.^10^ Community datasets were acquired from the original authors of those datasets.

### Gene Expression Levels

Illumina RNA-seq reads were downloaded from the NCBI SRA (BioProject PRJNA171684). Reads were trimmed of adaptors using Fastq-MCF^11^ trimmed for quality using DynamicTrim (Cox et al., 2010) and then aligned to the B73_RefGen_V3 and B73_RefGen_V4 genome assemblies using Hisat2 (Kim et al., 2019). We computed FPKM (Fragments Per Kilobase of transcript per Million mapped reads) and normalized read counts for each expression dataset for genes in each of the three *Z. mays* gene sets using cuffquant and cuffnorm, which are part the Cufflinks package (Trapnell et al., 2010).

### Gene Database Cross References

Mappings between AGPv3.21 and AGPv4 genes, which originated from the B73_RefGen_V3 and B73_RefGen_V4 genome assemblies, respectively, were downloaded from the Gramene ftp site (Release 54)^12^ (Tello-Ruiz et al., 2018). Cross references between AGPv4 and RefSeq, which both originated from the B73_RefGen_V4 assembly, were computed based on overlapping locations of coding exons.

### Loading and Configuring InterMine

MaizeMine was constructed using the open-source InterMine data warehouse system. InterMine has a core PostGreSQL data model that can be extended for custom datasets (Smith et al., 2012). The InterMine client side webapp is also highly customizable. For MaizeMine we extended the data model to accommodate gene expression levels with metadata, multiple assembly releases for a single organism, multiple gene sets per genome assembly, and gene cross references. We also modified the Regions Search code to enable selection of a genome assembly release. Code modifications are available on GitHub^13^.

## Data Availability Statement

Publicly available datasets were analyzed in this study. This data can be found here: https://www.ncbi.nlm.nih.gov/bioproject/171684.

## Author Contributions

MS, JG, and CE wrote the initial draft. MS, JG, DT, AT, and CE collected, curated, and formatted the data. MS, AW, JL, AT, DU, and HN performed code development, database builds, and webapp configuration. JP and JT integrated MaizeMine with MaizeGDB. MS, AW, and CE developed the examples. JG and CE performed the outreach. JG, DT, JP, EC, and CA tested MaizeMine and the use examples. CE and CA provided the project administration. CA was responsible for funding acquisition. All authors were involved in reviewing and editing of the manuscript.

## Conflict of Interest

The authors declare that the research was conducted in the absence of any commercial or financial relationships that could be construed as a potential conflict of interest.

## Footnotes

1 http://www.maizegdb.org

2 http://mines.legumeinfo.org/

3 https://maizemine.maizegdb.org/

4 https://intermine.readthedocs.io/en/latest/web-services/

5 https://bigd.big.ac.cn/gwas/

6 https://nph.onlinelibrary.wiley.com/action/downloadSupplement?doi=10.1111%2Fnph.14893&file=nph14893-sup-0002-NotesS1-S5.xlsx

7 https://github.com/elsiklab/

8 https://www.maizegdb.org/assembly#downloads

9 https://ftp.ncbi.nlm.nih.gov/genomes/refseq/plant/Zea_mays/all_assembly_versions/suppressed/GCF_000005005.2_B73_RefGen_v4/

10 https://maizemine.maizegdb.org/dataCategories.do

11 https://expressionanalysis.github.io/ea-utils/

12 ftp://ftp.gramene.org/pub/gramene/archives/PAST_RELEASES/release-54/gff3/zea_mays/gene_id_mapping_v3_to_v4/

13 https://github.com/elsiklab/

## Supplementary Material

Supplementary Table 1 Chromosome coordinates to use as input for Example 1.

Supplementary Table 2 Gene set enrichment results from Example 2. Each worksheet corresponds to one enrichment test, and is labeled according to the gene set and enrichment dataset (e.g., Gene Ontology or pathway dataset).

Supplementary Data Sheet 1 Supplementary Figures 1–19.

Supplementary Data Sheet 2 API examples.
