# Supplementary material for: MaizeMine: A Data Mining Warehouse for the Maize Genetics and Genomics Database
Source: Front Plant Sci. 2020 Oct 22;11:592730. doi: 10.3389/fpls.2020.592730 (PMC7642280; doi:10.3389/fpls.2020.592730)
Supplement: Supplementary Data Sheet 1 — Supplementary Figures 1–19. [file Data_Sheet_1.PDF]

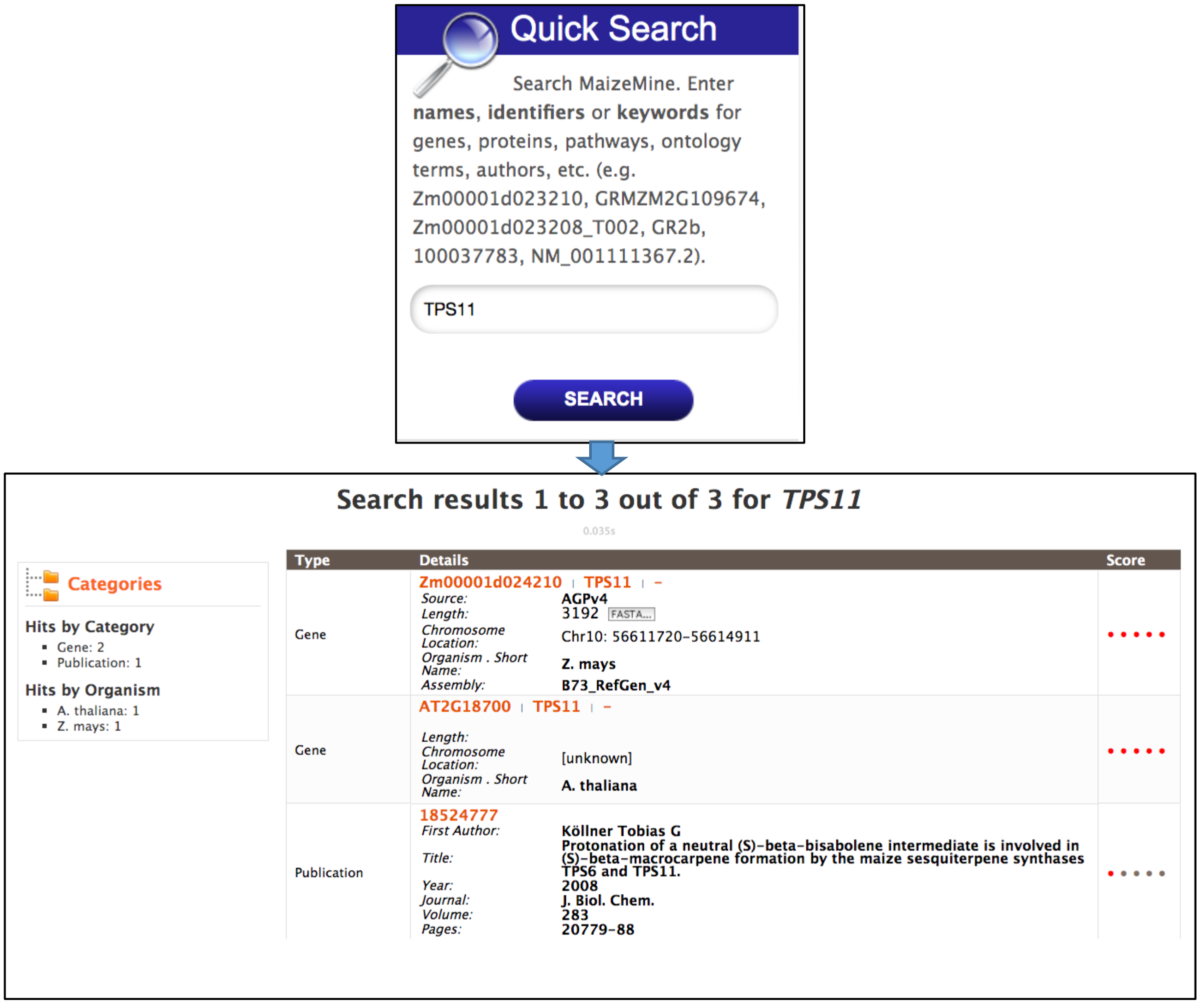

Supplementary Figure 1. Quick Search menu and output. The Quick Search performs a full text search of the MaizeMine data records, provides a list of results that can be filtered by data type ('Category') and organism. Clicking an identifier in the output list leads to a report page.

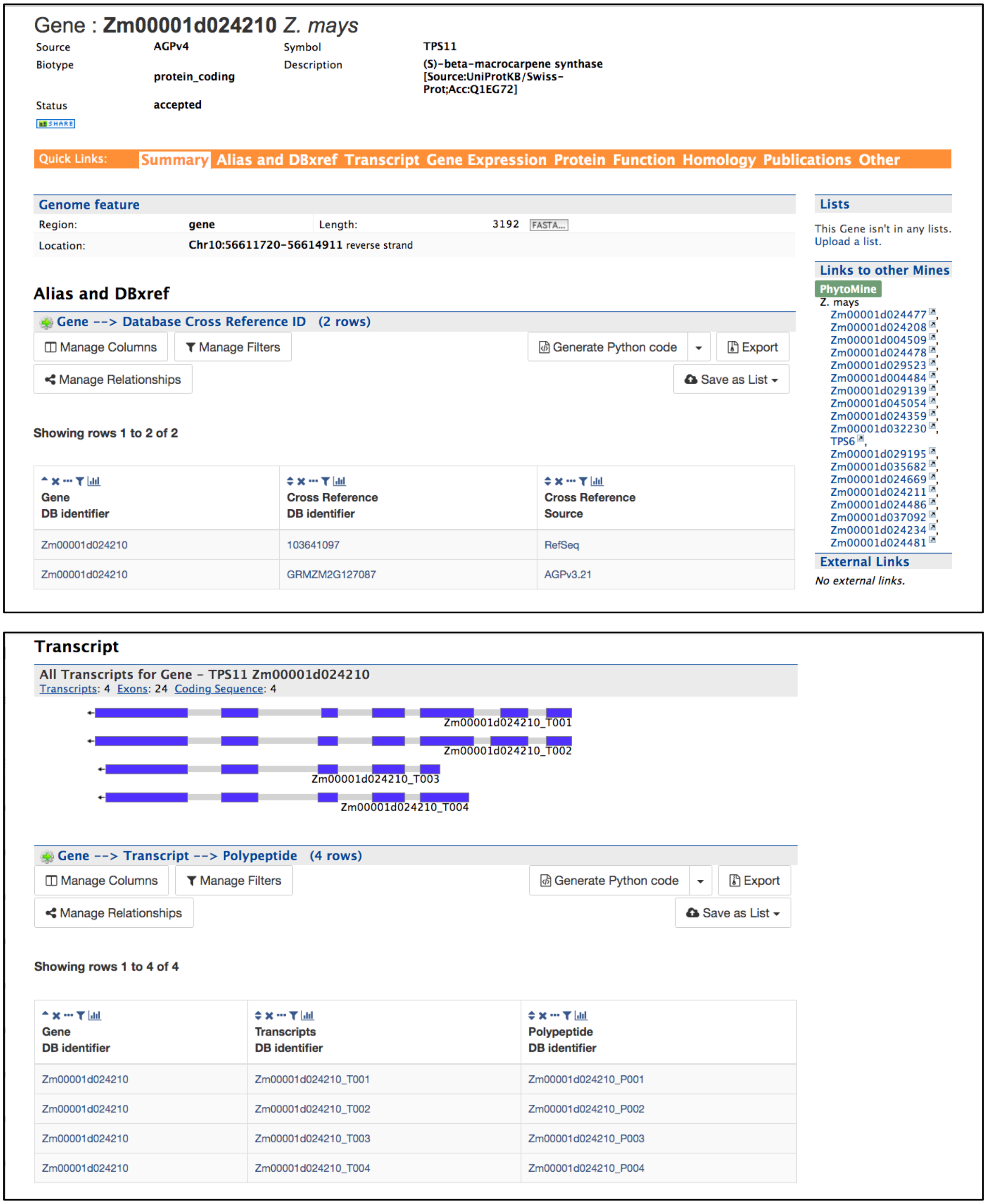

Supplementary Figure 2. Upper part of Gene Report, showing the Summary, Alias and DBxref, and Transcript sections.

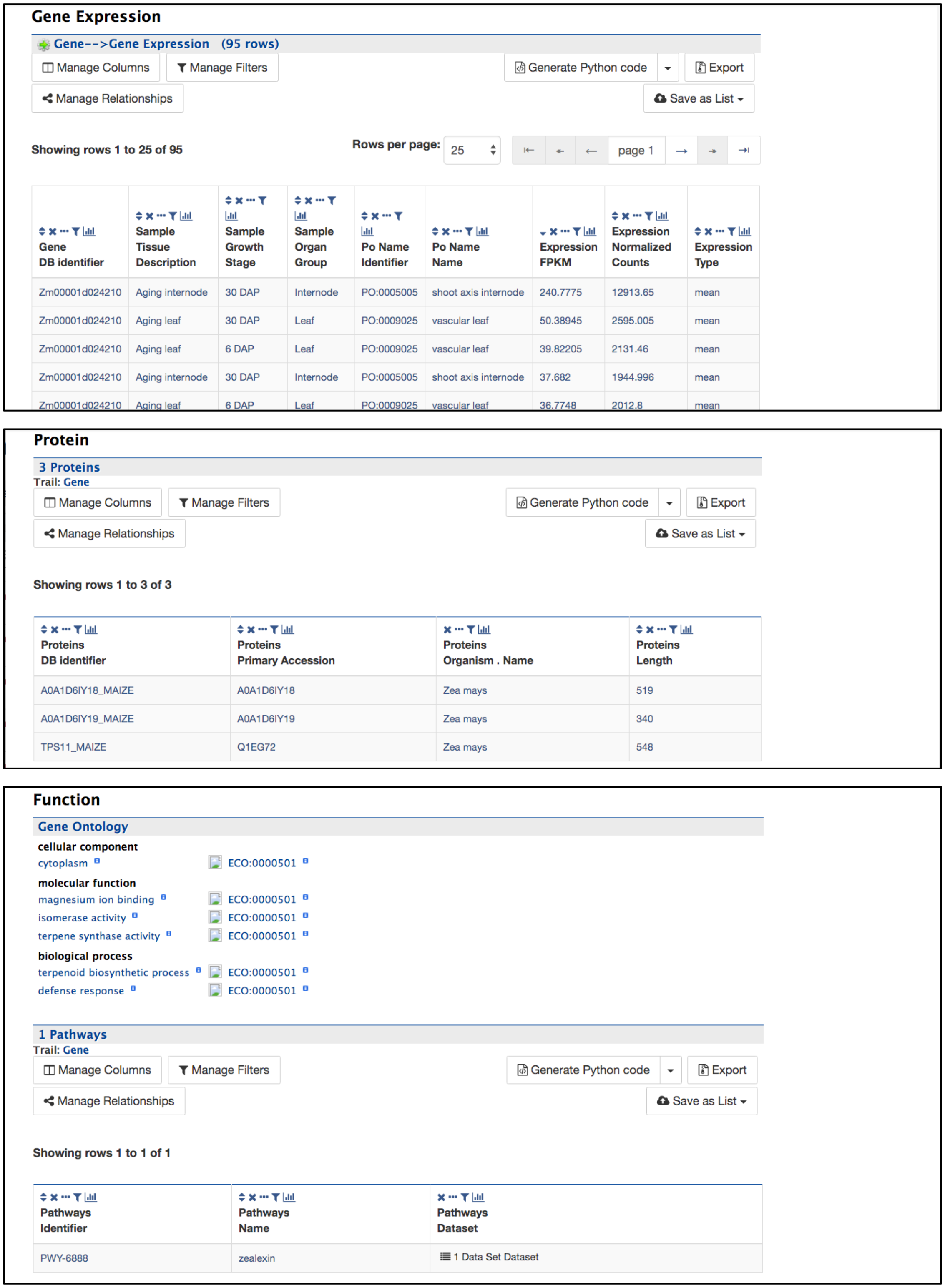

Supplementary Figure 3. Middle part of a Gene Report, showing the Gene Expression, Proteins, and Function sections.

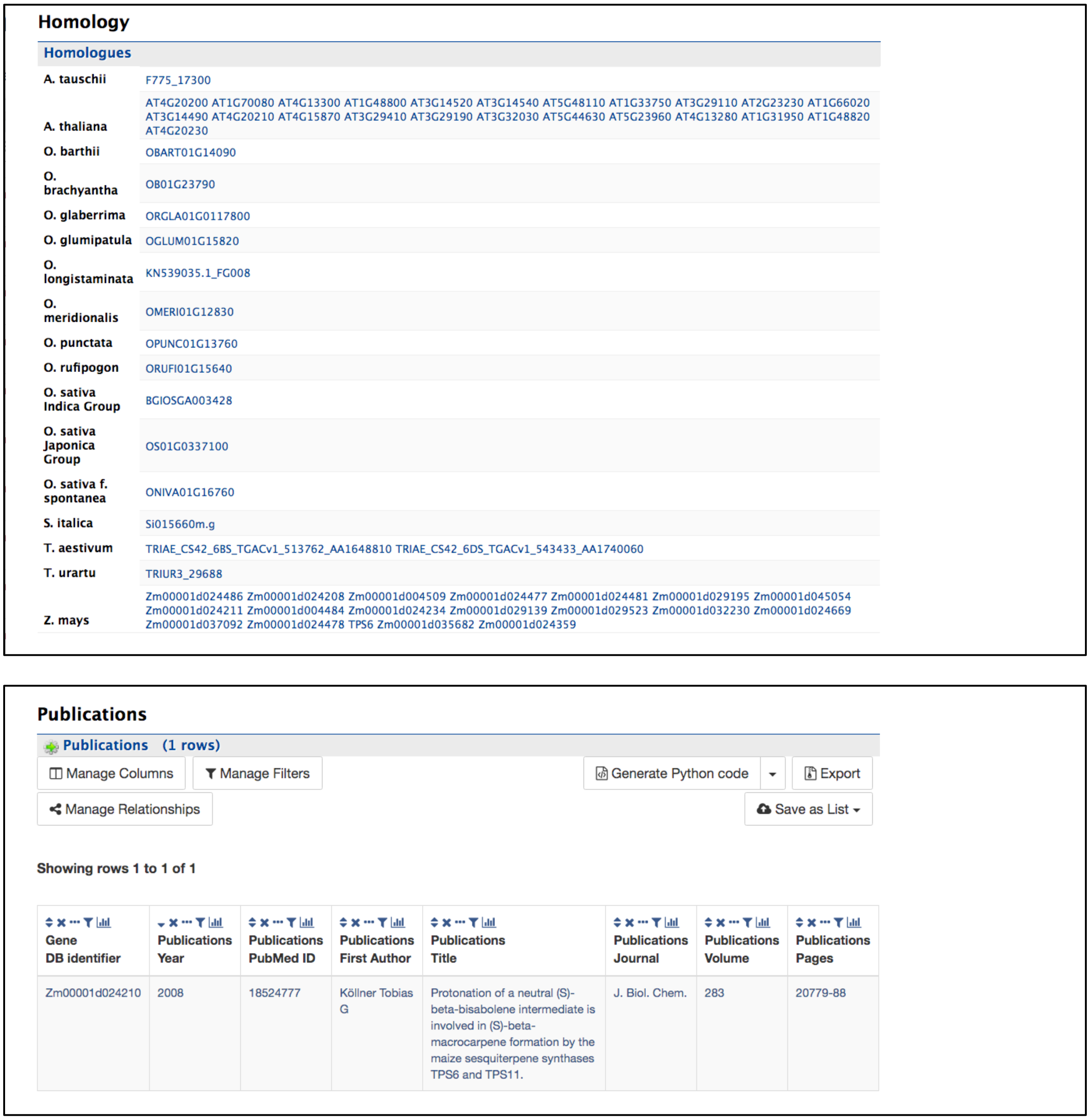

Supplementary Figure 4. Lower part of a Gene Report, showing the Homology and Publication sections.

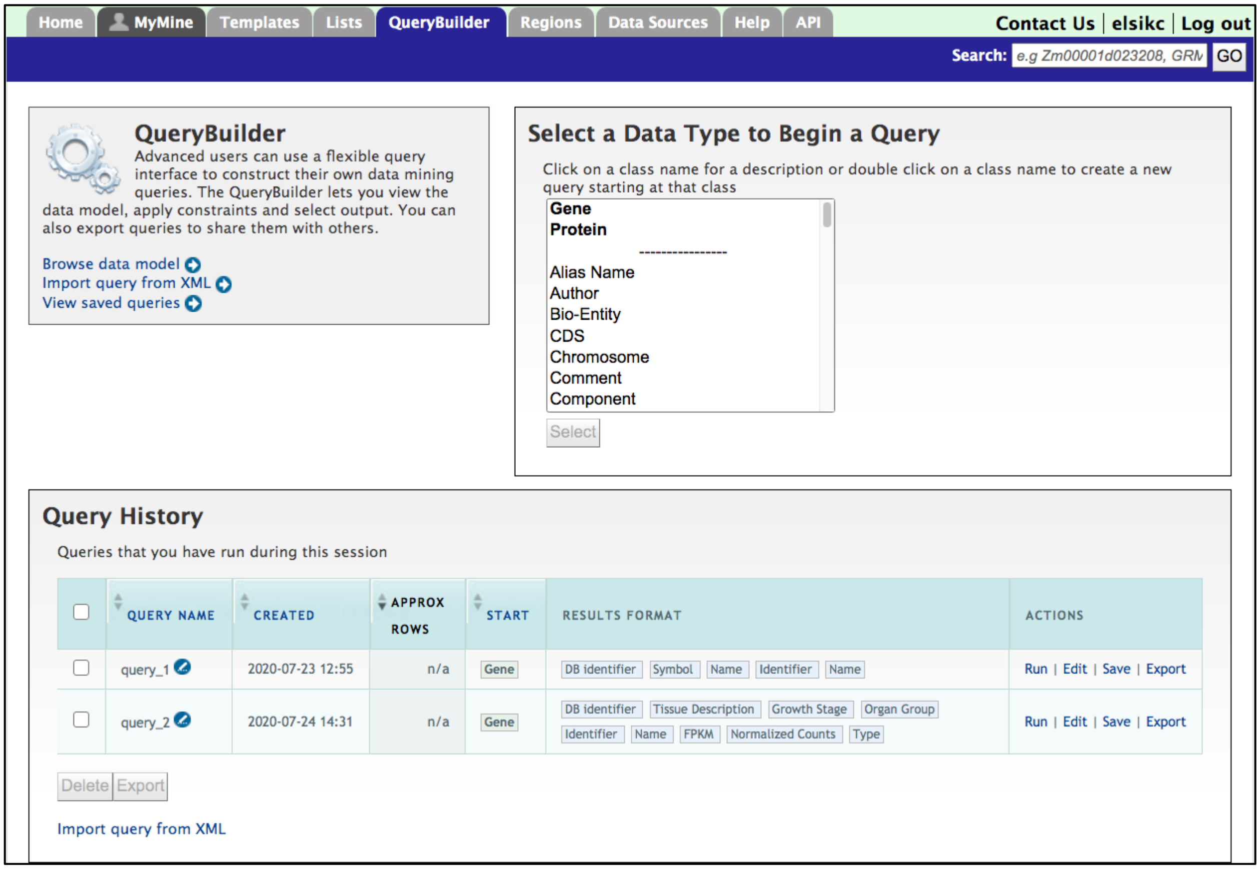

Supplementary Figure 5. QueryBuilder page. The QueryBuilder box in the upper left corner includes a link to 'Browse the data model', which leads to the data tree shown in Figure S6. It also provides a link that allows you to import a query XML that you have saved locally. The Query History box shows recent queries, and allows you to rerun, edit, save or export the query. The box 'Select a Data Type to Begin a Query' provides the easiest way to access the Model Browser and Query Overview (shown in Figure 4), to start construction of a new query.

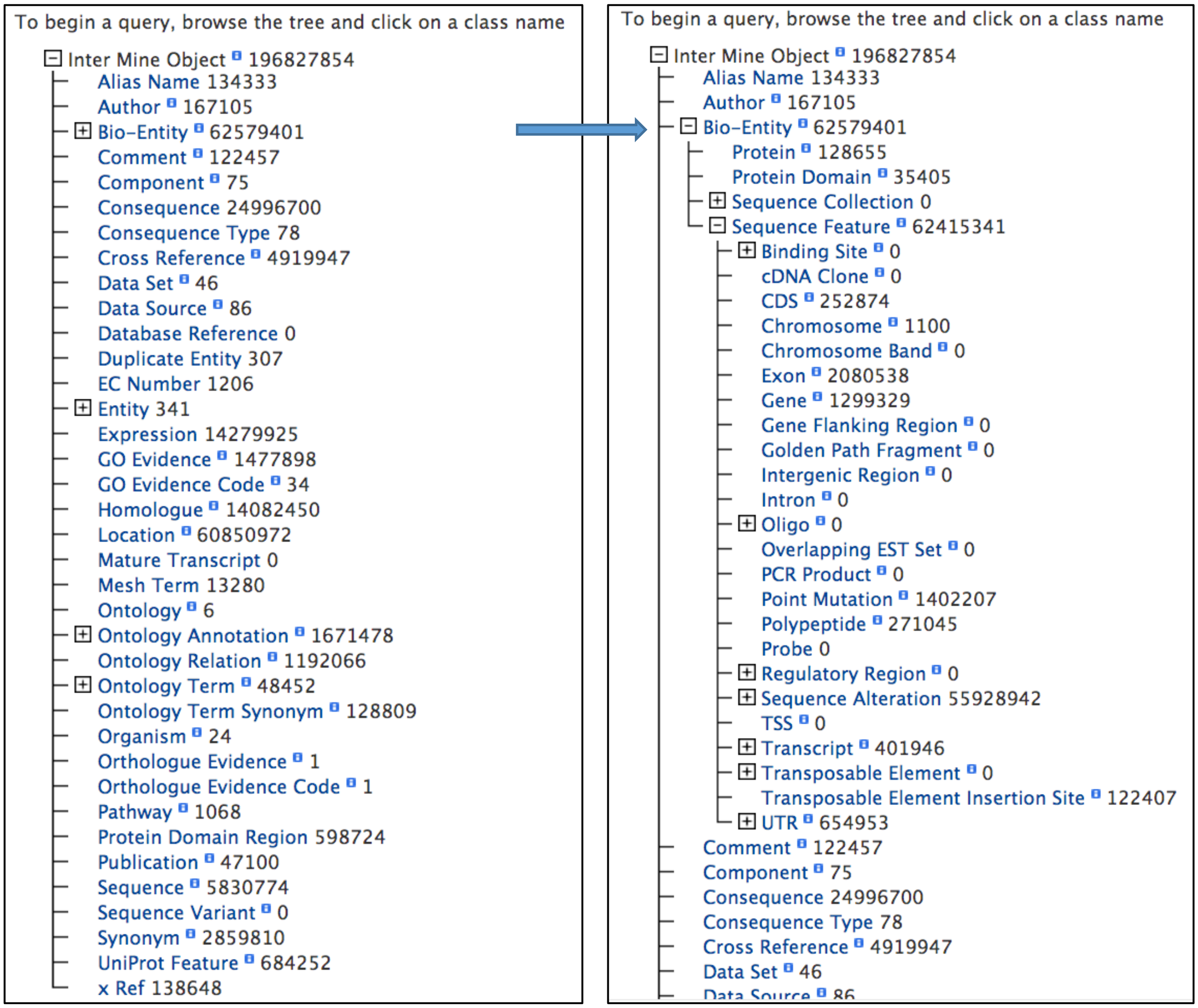

Supplementary Figure 6. MaizeMine data tree. This shows the hierarchical data structure of MaizeMine, with the numbers of each entity in the database. The panel on the right shows Bio-Entity and Sequence Feature expanded. Clicking and entity in the tree opens the Model Browser and Query Overview (Figure 4).

frontiers

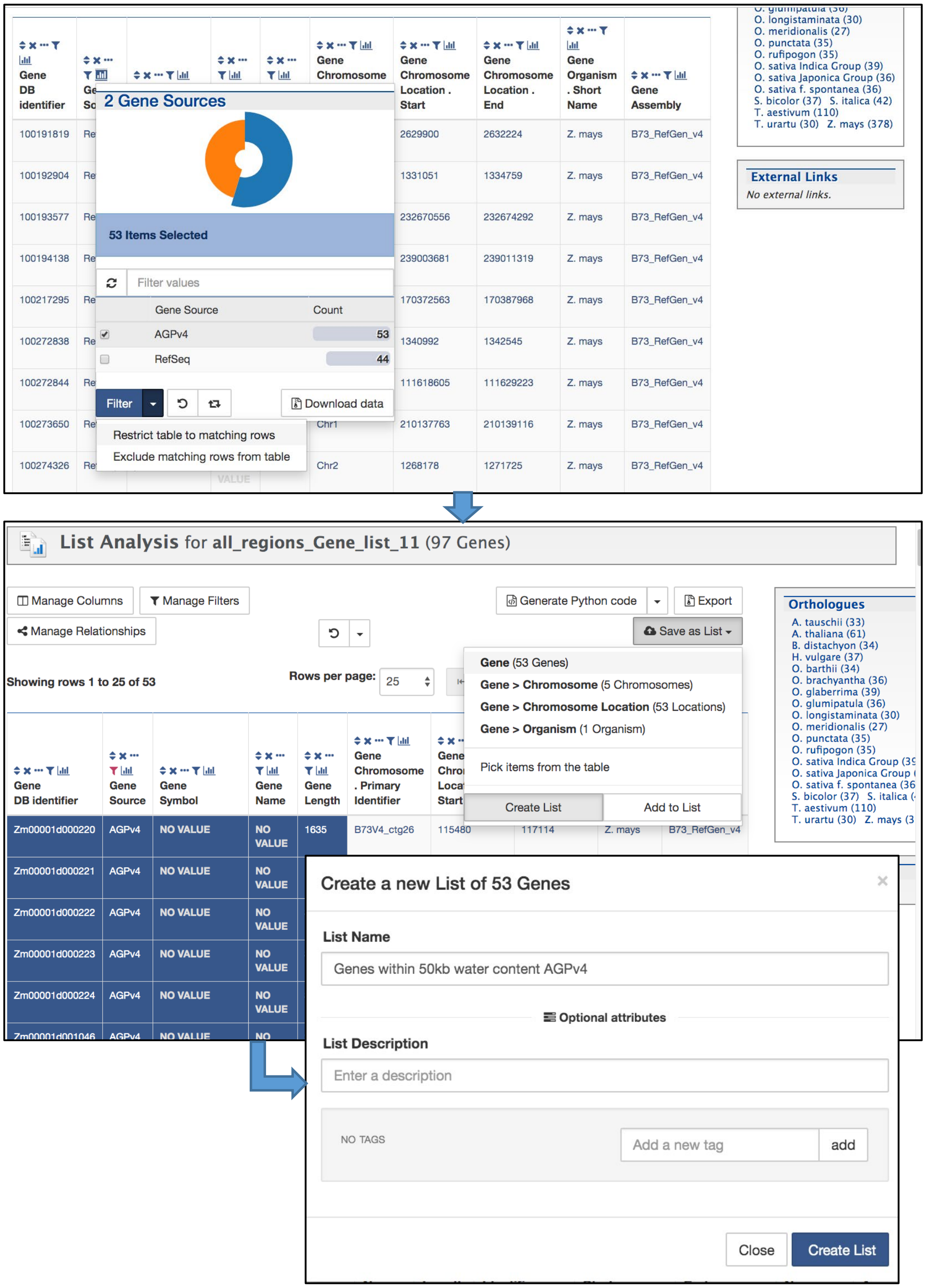

Supplementary Figure 7. Gene list saved after doing the Regions search in Example 1. Both AGPv4 and RefSeq genes were returned in the search. The histogram icon above the Gene Source column is used to filter for AGPv4 genes. Then a new list of only AGPv4 genes is saved. An undo button above the table (not shown) allows you to undo the filtering step, so that you can filter and save RefSeq genes.

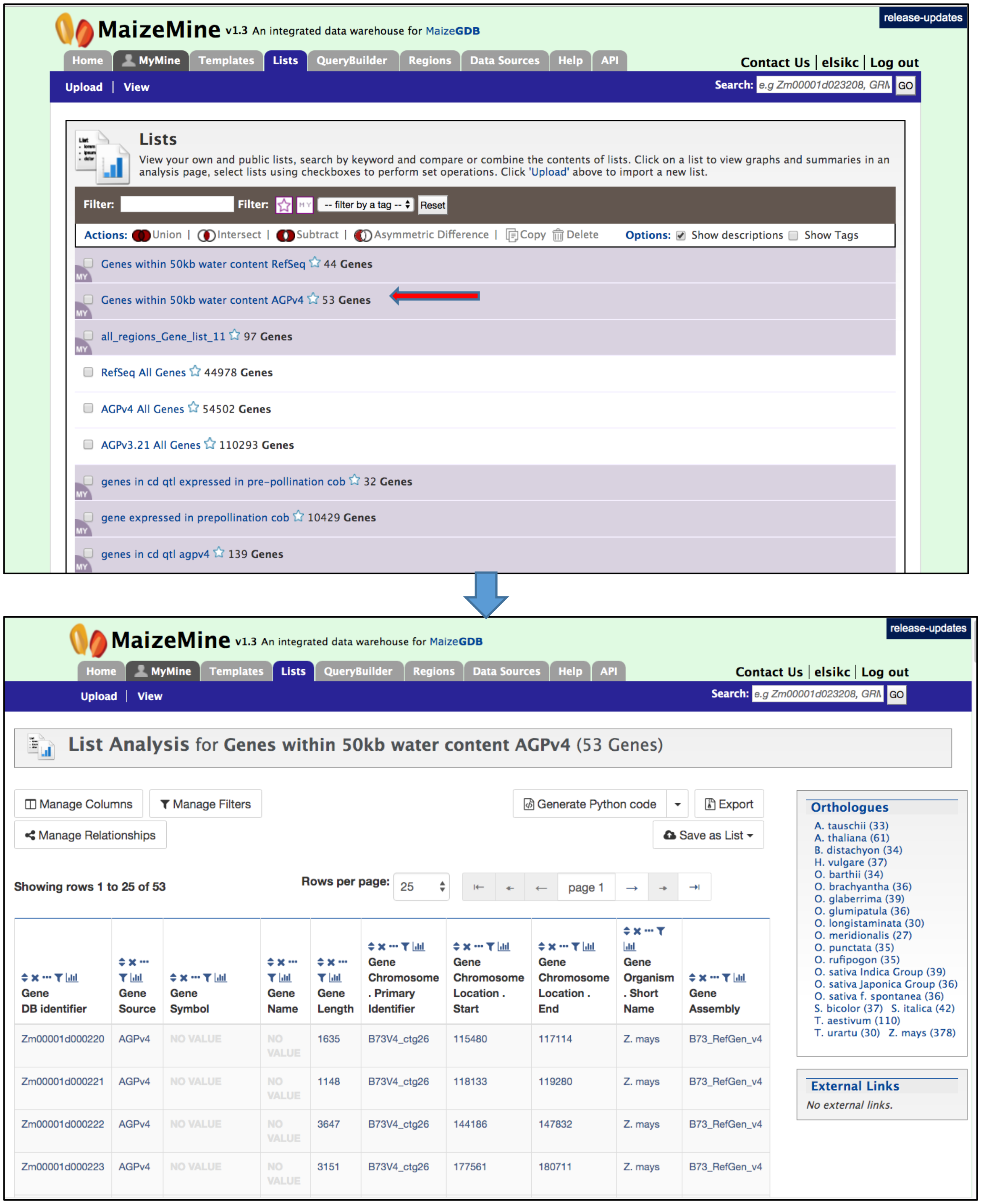

Supplementary Figure 8. List View and Analysis pages. The top panel shows the List View page after saving the AGPv4 and RefSeq gene lists within the searched regions from Example 1. User-saved lists are shown with a mauve background and default lists available to all users are shown with a white background. Clicking the AGPv4 list name leads to the List Analysis page. Below the table are the gene enrichment widgets. Notice the blue bar below the main navigation bar provides 'Upload' and 'View' which allow you to toggle to the List Upload or List View page, respectively.

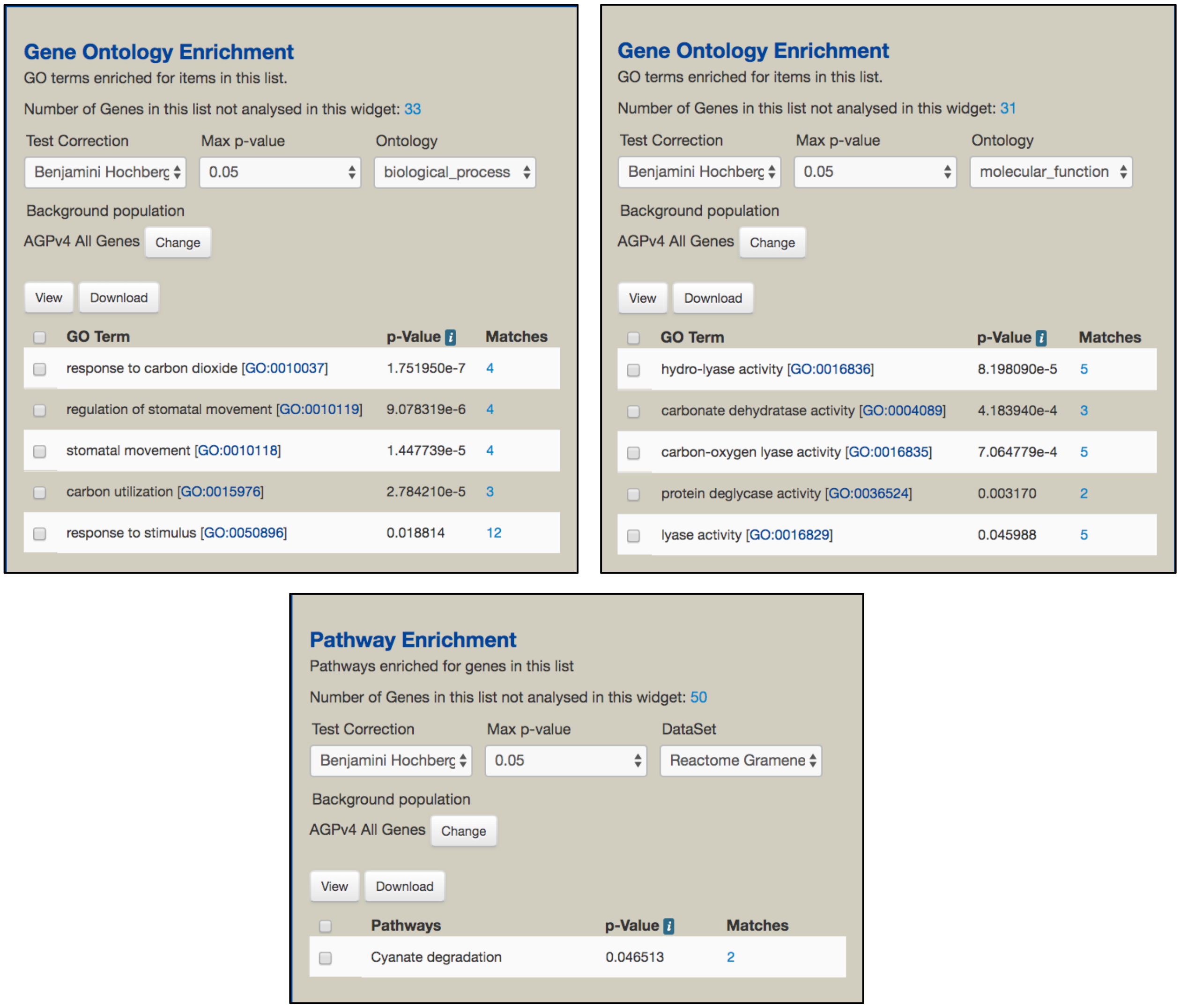

Supplementary Figure 9. Enrichment widgets showing GO and Reactome pathway enrichment for the AGPv4 genes identified in the regions search in Example 1. The background population has been changed to 'AGPv4 All Genes' and test correction has been changed to 'Benjamini Hochberg' in each of the widgets. Results can be downloaded as tab delimited files using the Download buttons.

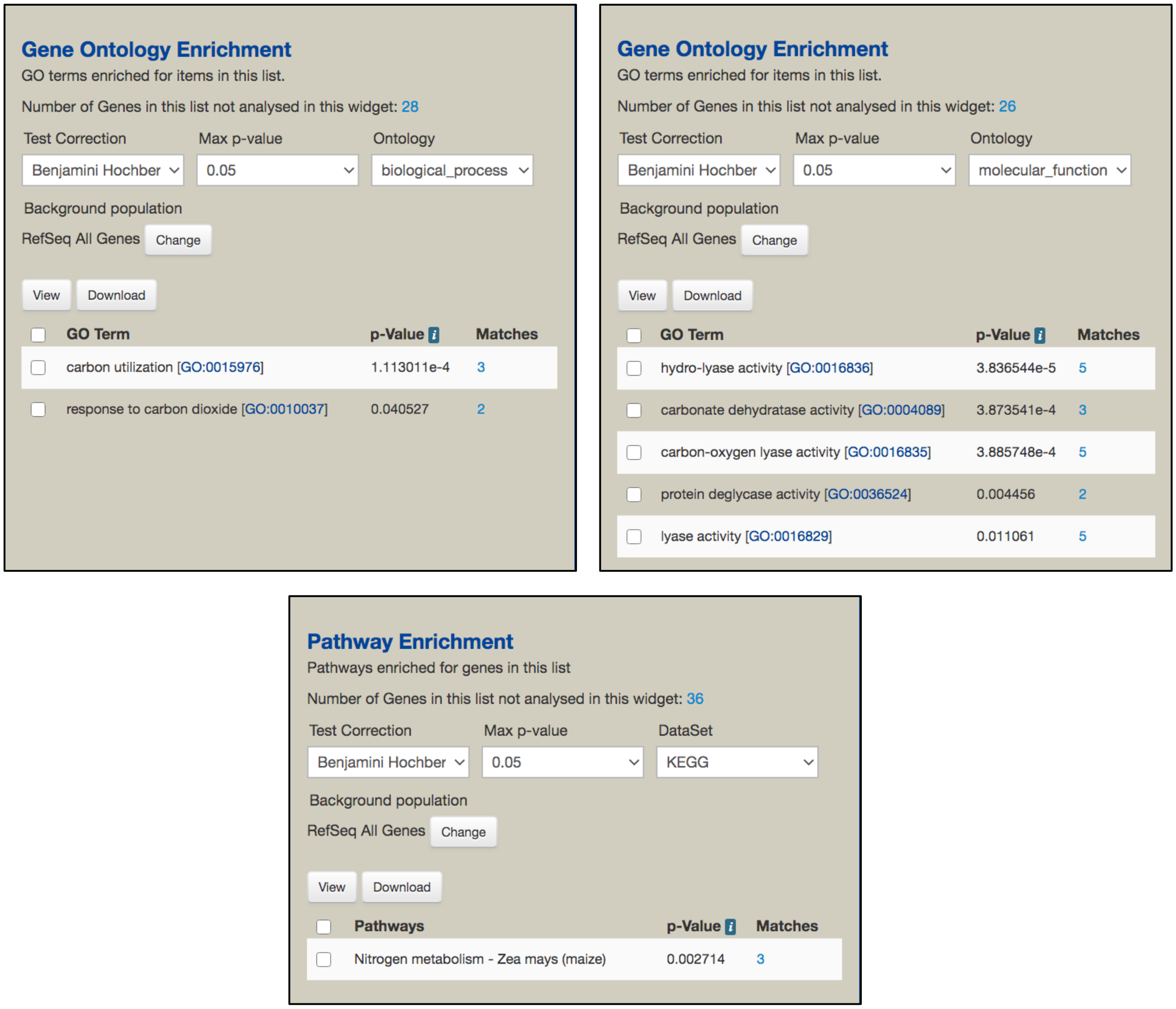

Supplementary Figure 10. Enrichment widgets showing GO and KEGG pathway enrichment for the RefSeq genes identified in the regions search in Example 1. The background population has been changed to 'RefSeq All Genes' and test correction has been changed to 'Benjamini Hochberg' in each of the widgets.

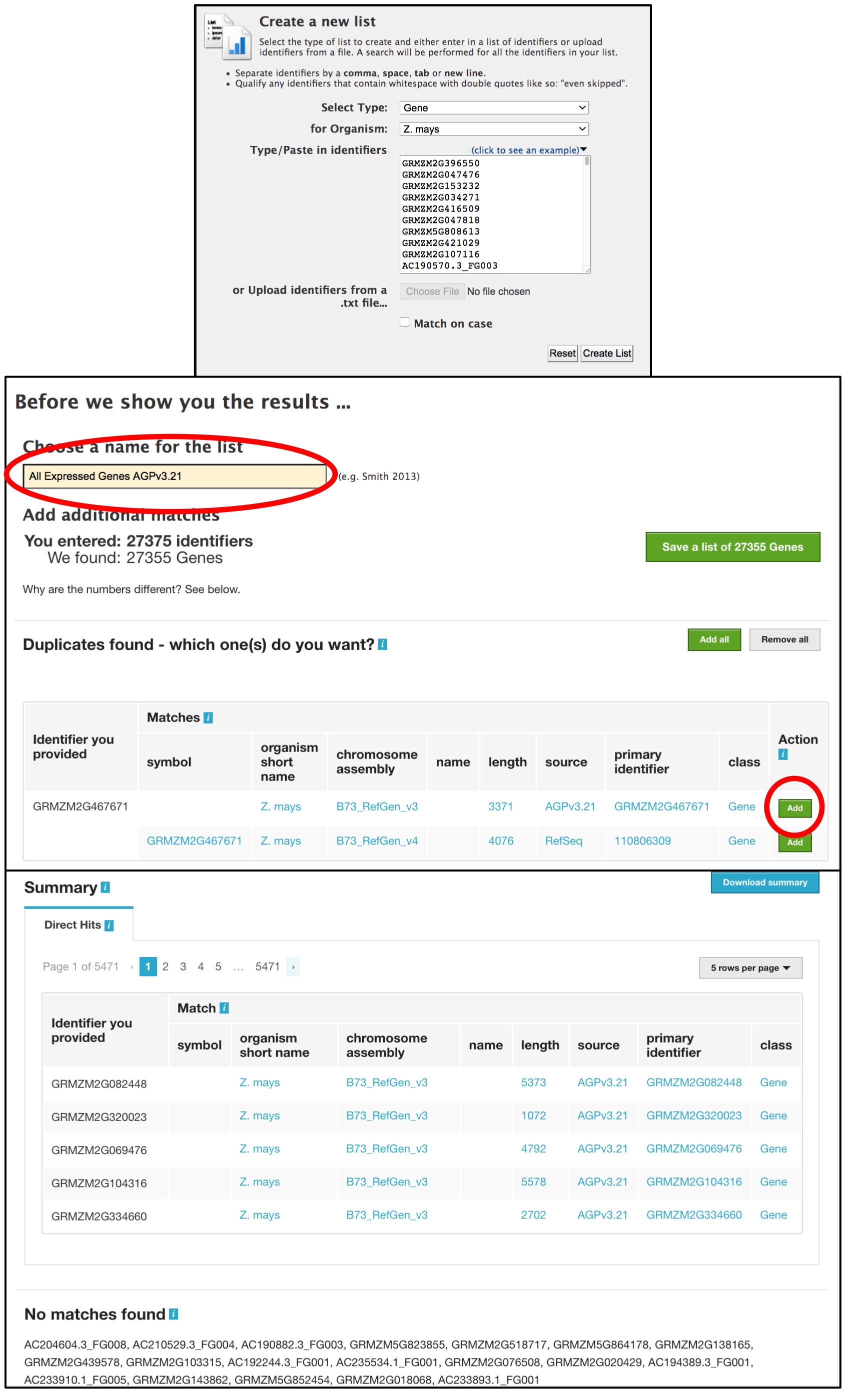

Supplementary Figure 11. Uploading the background gene list for Example 2. After clicking ‘Create List’ in the upload menu, a database lookup is performed to verify presence of the identifiers in MaizeMine and finds that some ids are missing. The missing ids are listed at the bottom of the page. You are prompted to name the list. One id is duplicated, and of those duplicates we selected the AGPv3.21 gene.

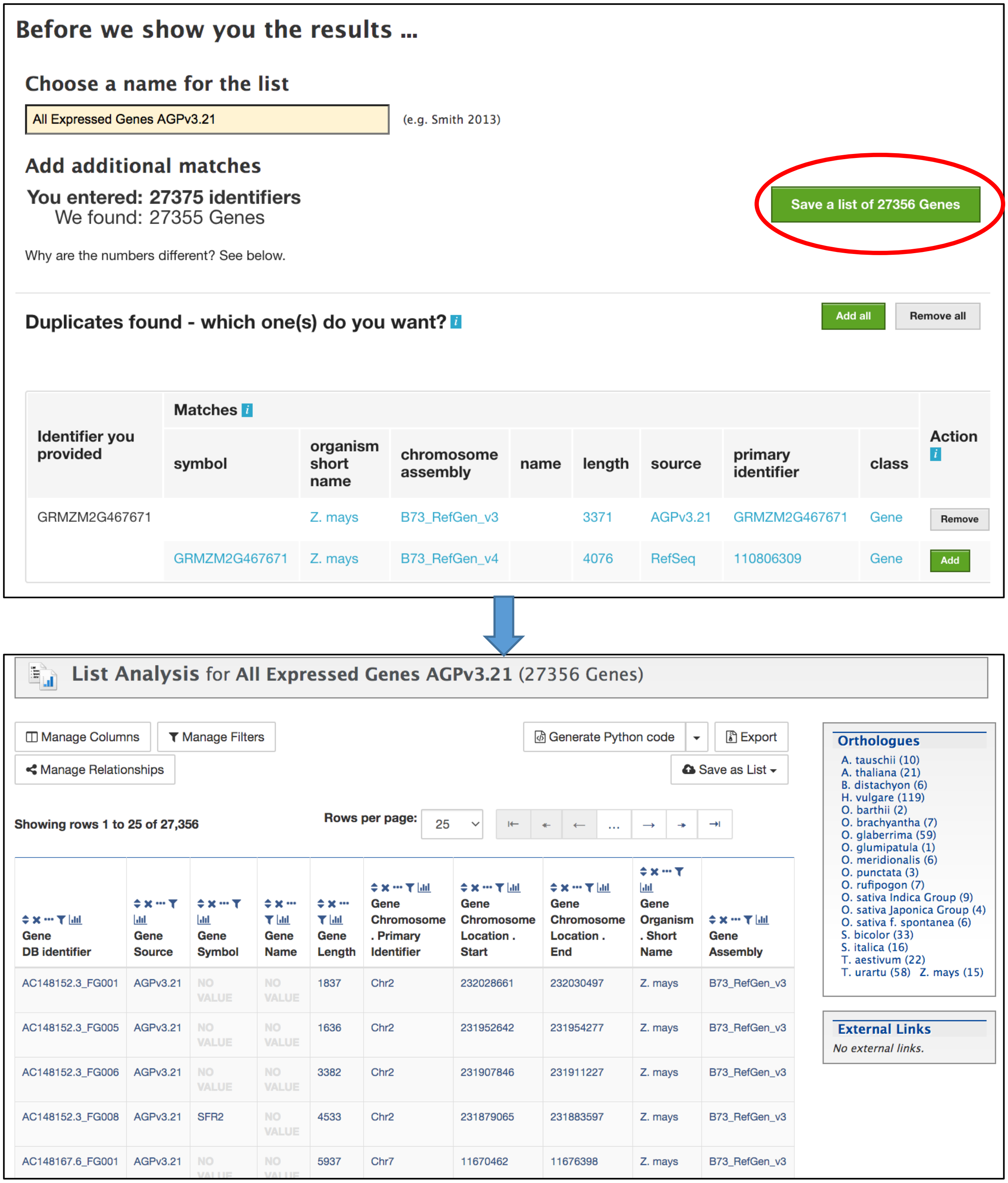

Supplementary Figure 12. Finishing the process of saving the background gene list for Example 2. One of the duplicated ids has been selected and added to the final list. The final step is to ‘Save a list of 27356 Genes’, which opens the List Analysis page showing a table of genes with default information. At this point the list is saved in the user account and will appear on the List View page (similar to the top of Supplementary Figure 8).

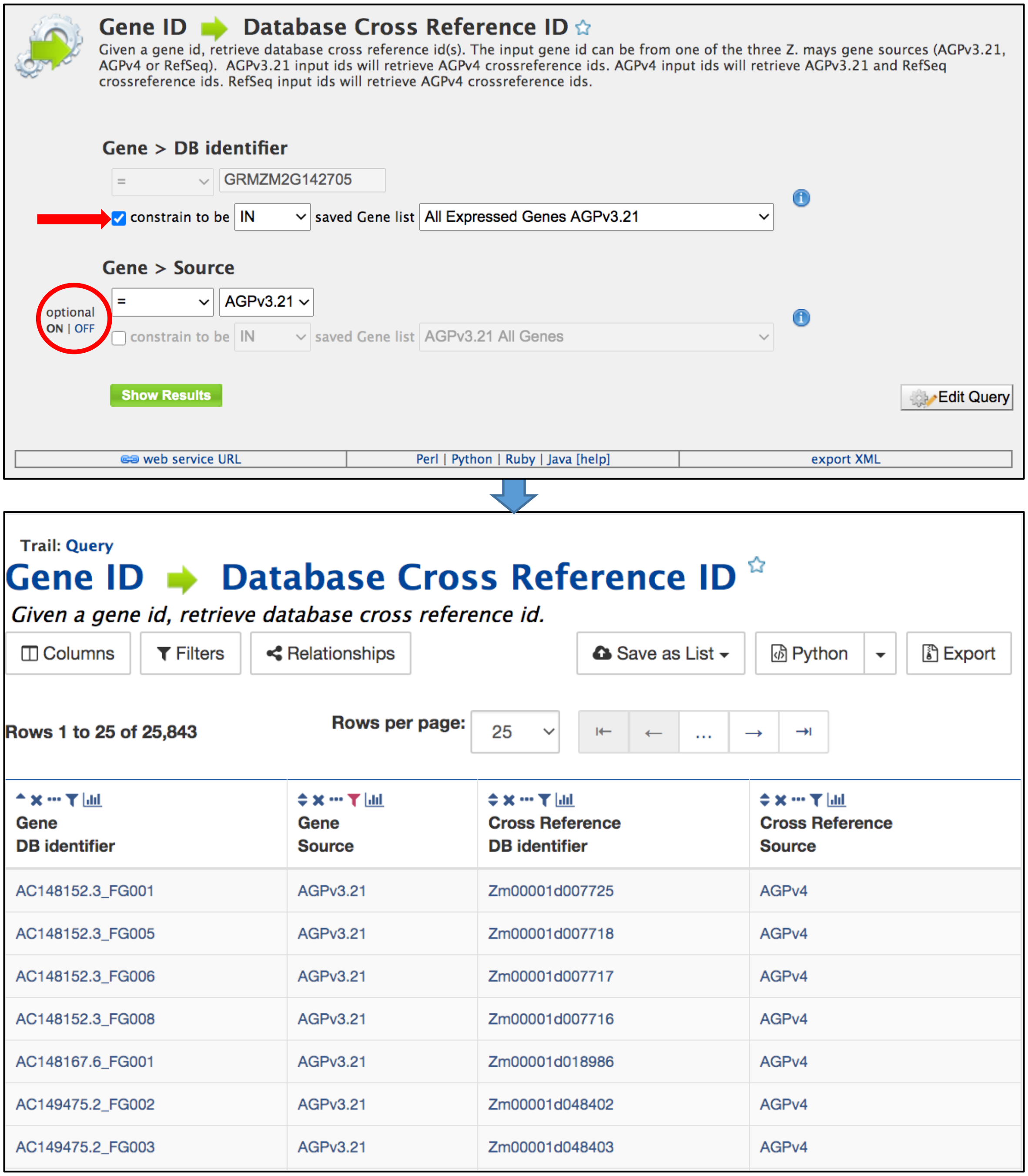

Supplementary Figure 13. Using a template query to retrieve database cross references for the AGPv3.21 background gene list saved in Example 2. Clicking the box next to ‘constrain to be in’ activates a pulldown menu where you can select the saved gene list. The optional Gene Source constraint is turned on, to limit the search to AGPv3.21 genes, because some of the input gene ids are found in more than one gene set. The output shows cross references to AGPv4 gene ids.

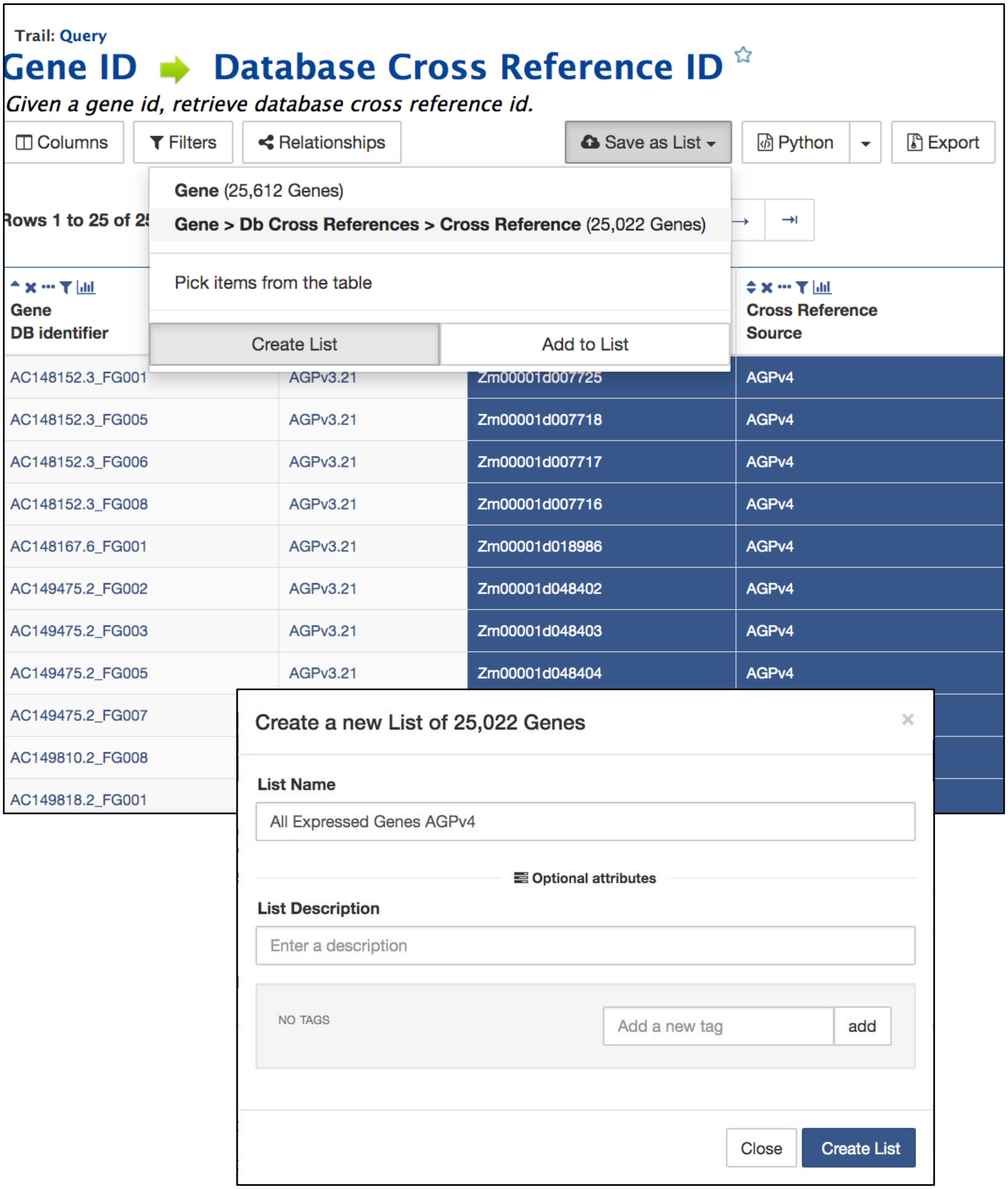

Supplementary Figure 14. Saving a list of AGPv4 cross reference gene ids from a table output. Clicking 'Save as List' above the table allows you to save a list of ids from a highlighted column. A menu opens allowing you to name the new list. After you click 'Create List', the new list is available on your List View page.

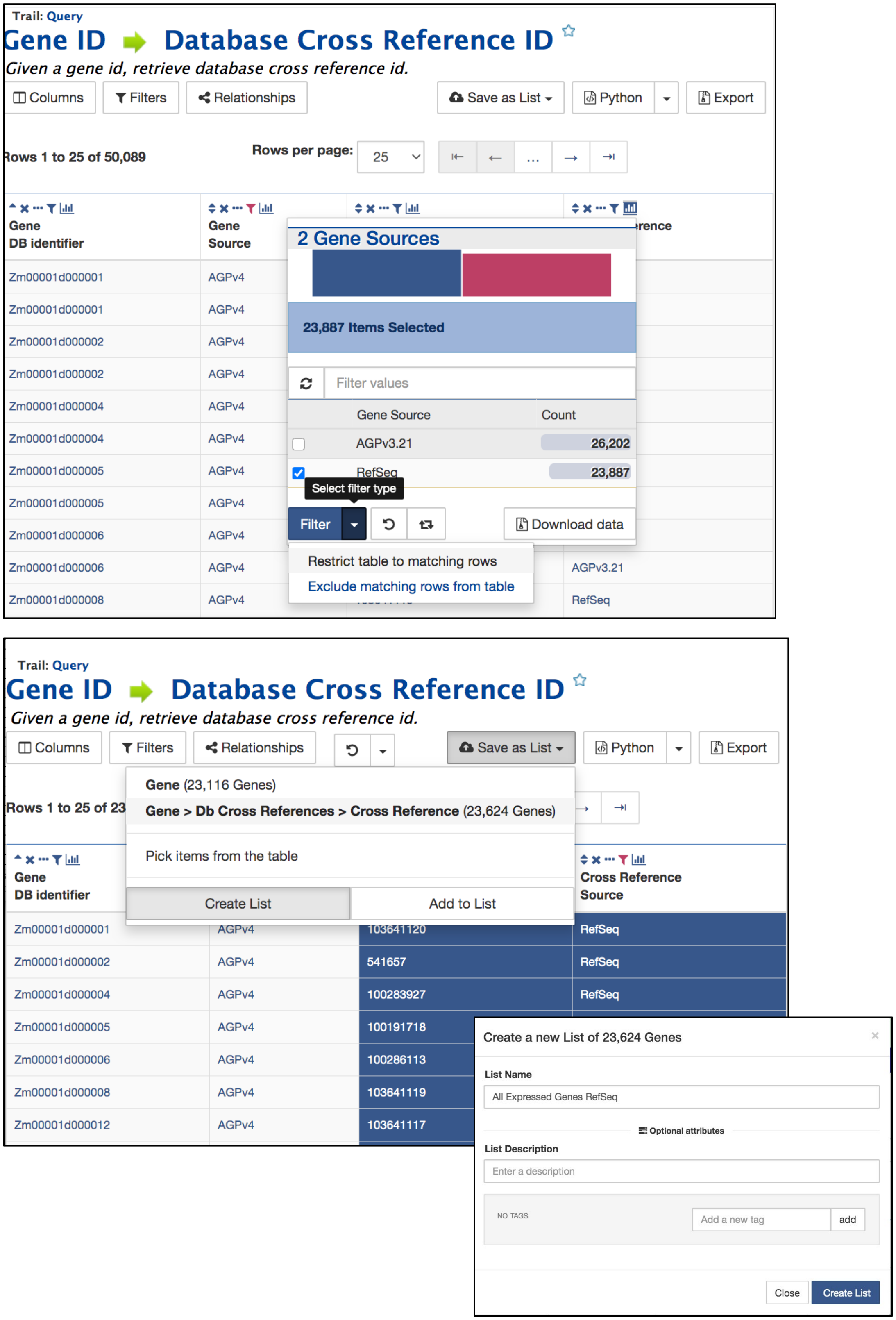

Supplementary Figure 15. Saving RefSeq database cross reference ids for the AGPv4 genes in Example 2. Because AGPv4 genes are cross-referenced to both AGPv3.21 and RefSeq ids, the table must be filtered for RefSeq ids prior to saving the new RefSeq gene list.

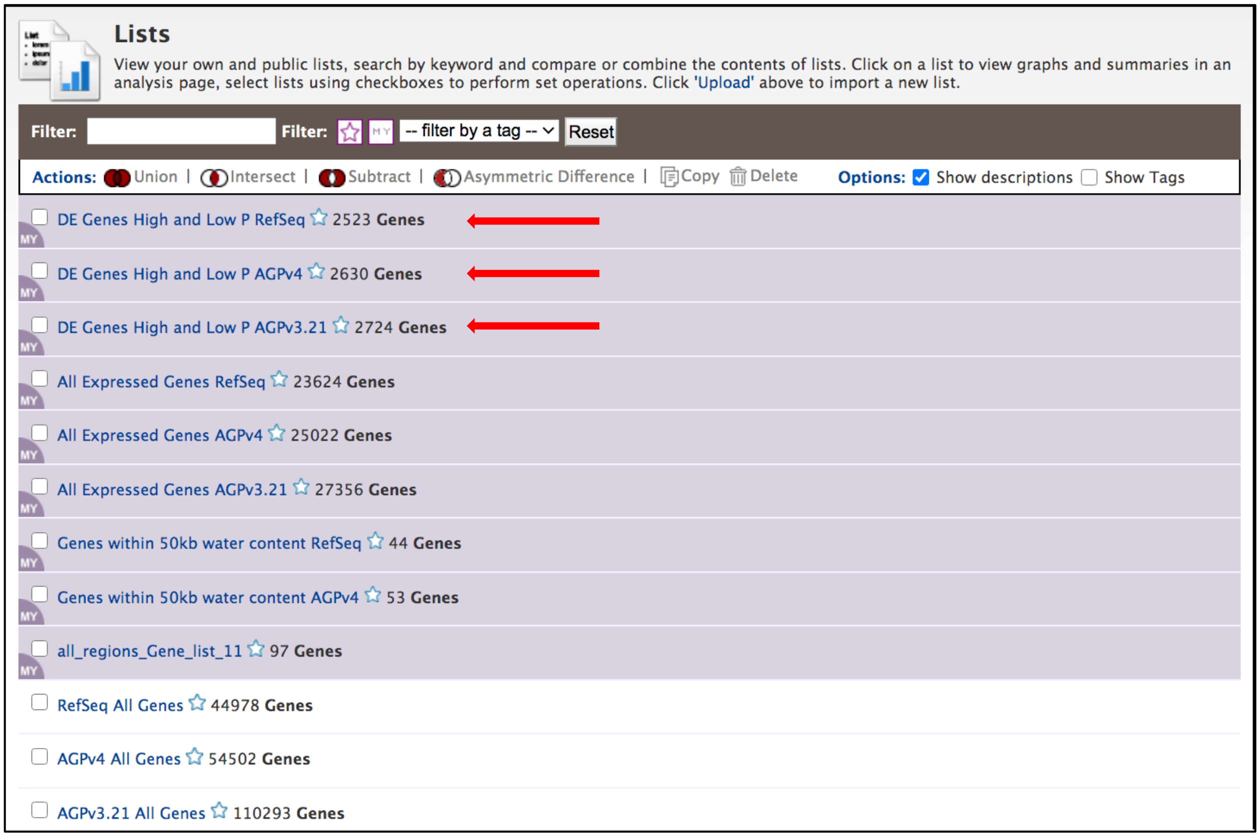

Supplementary Figure 16. The List View Page after saving all the lists in Example 2. Each of the lists of differentially expressed genes is analyzed separately by clicking the list name, which opens the List Analysis page and automatically activates the enrichment widgets.

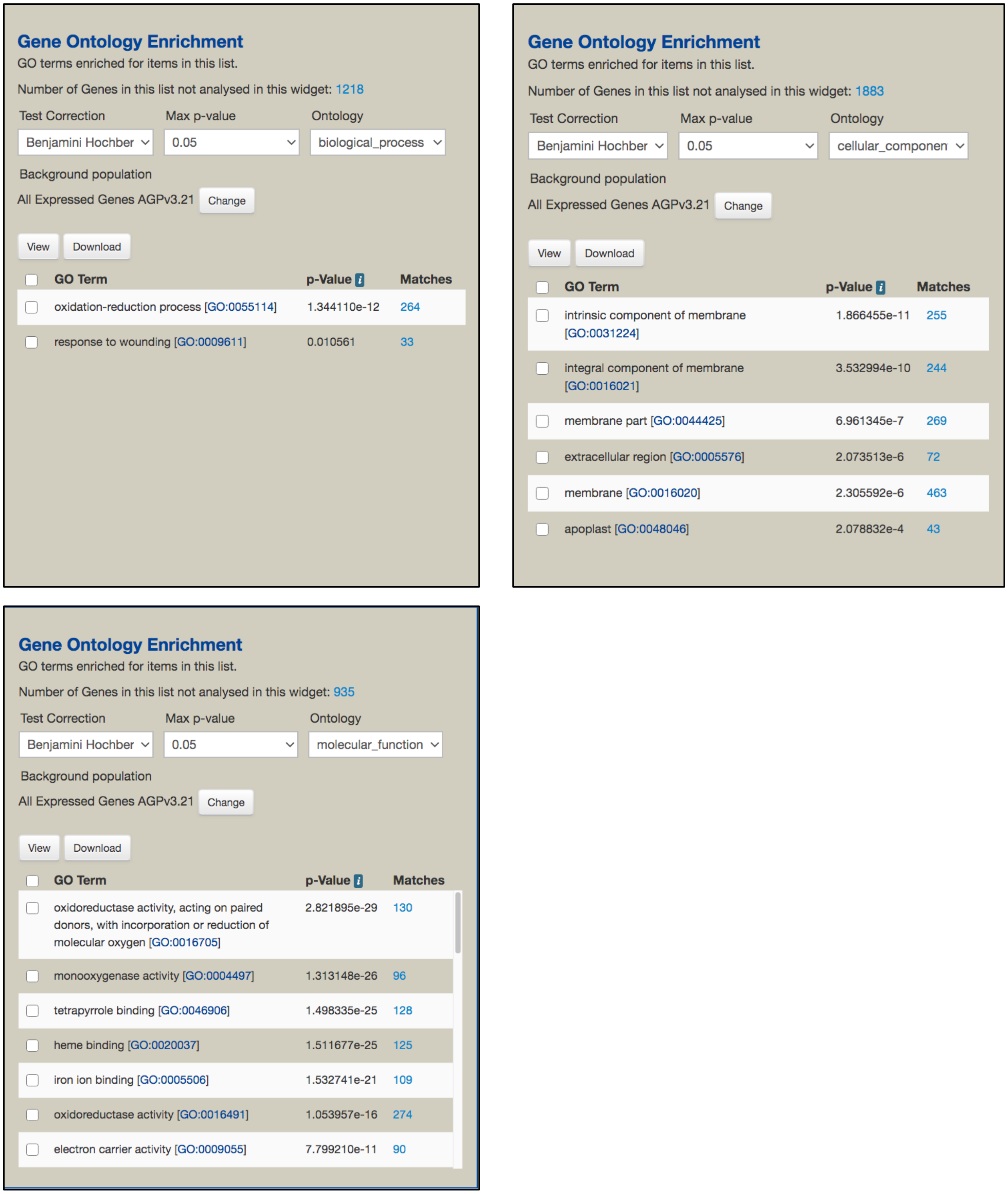

Supplementary Figure 17. Enrichment of GO terms for 'DE Genes High and Low P AGPv3.21' in Example 2. The background population has been changed to 'All Expressed Genes AGPv3.21' and text correction has been changed to 'Benjamini Hochberg'.

frontiers

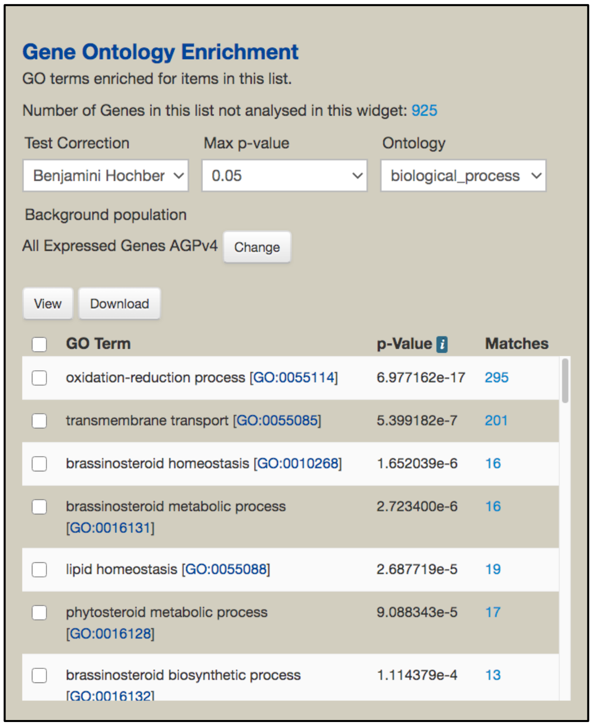

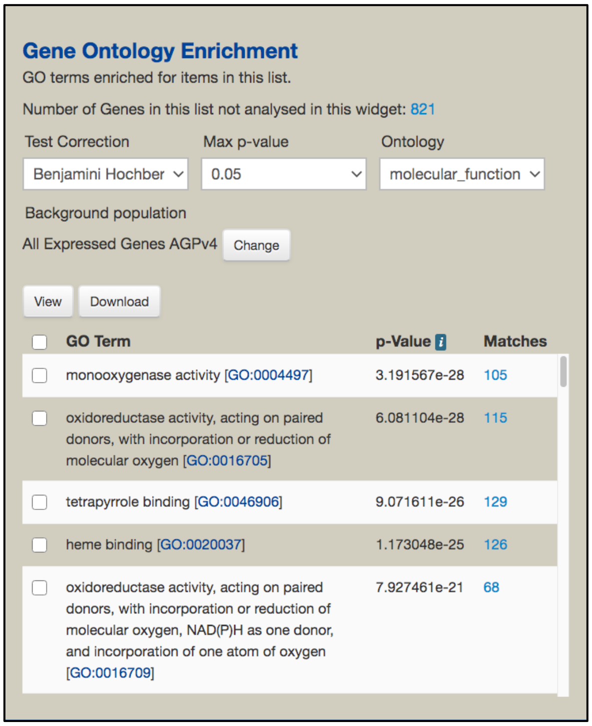

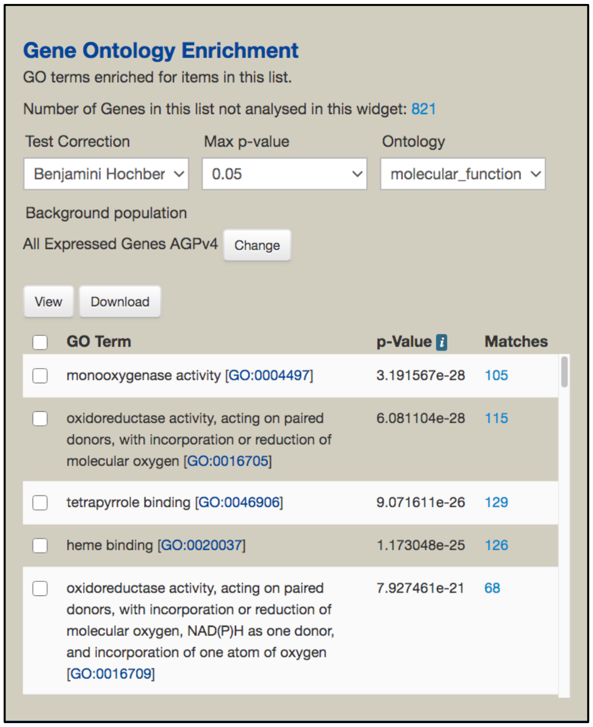

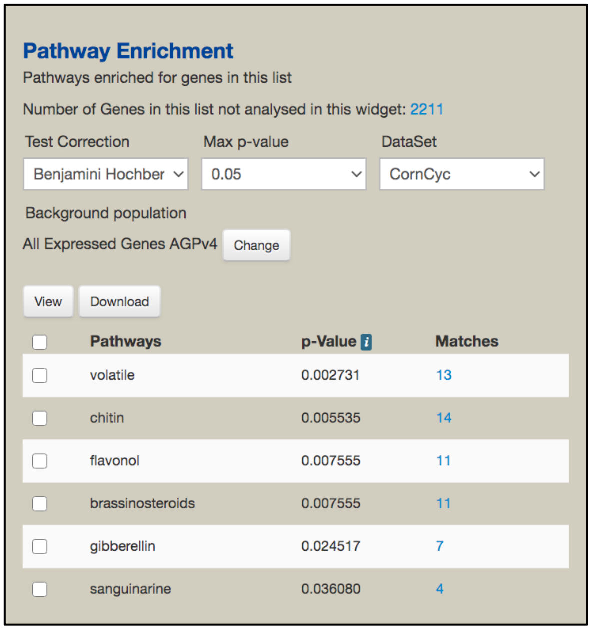

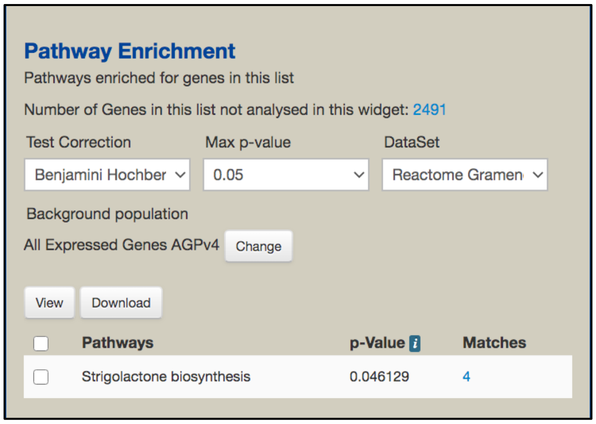

Supplementary Figure 18. Enrichment of GO and pathway terms for 'DE Genes High and Low P AGPv4' in Example 2. The background population has been changed to 'All Expressed Genes AGPv4' and text correction has been changed to 'Benjamini Hochberg'.

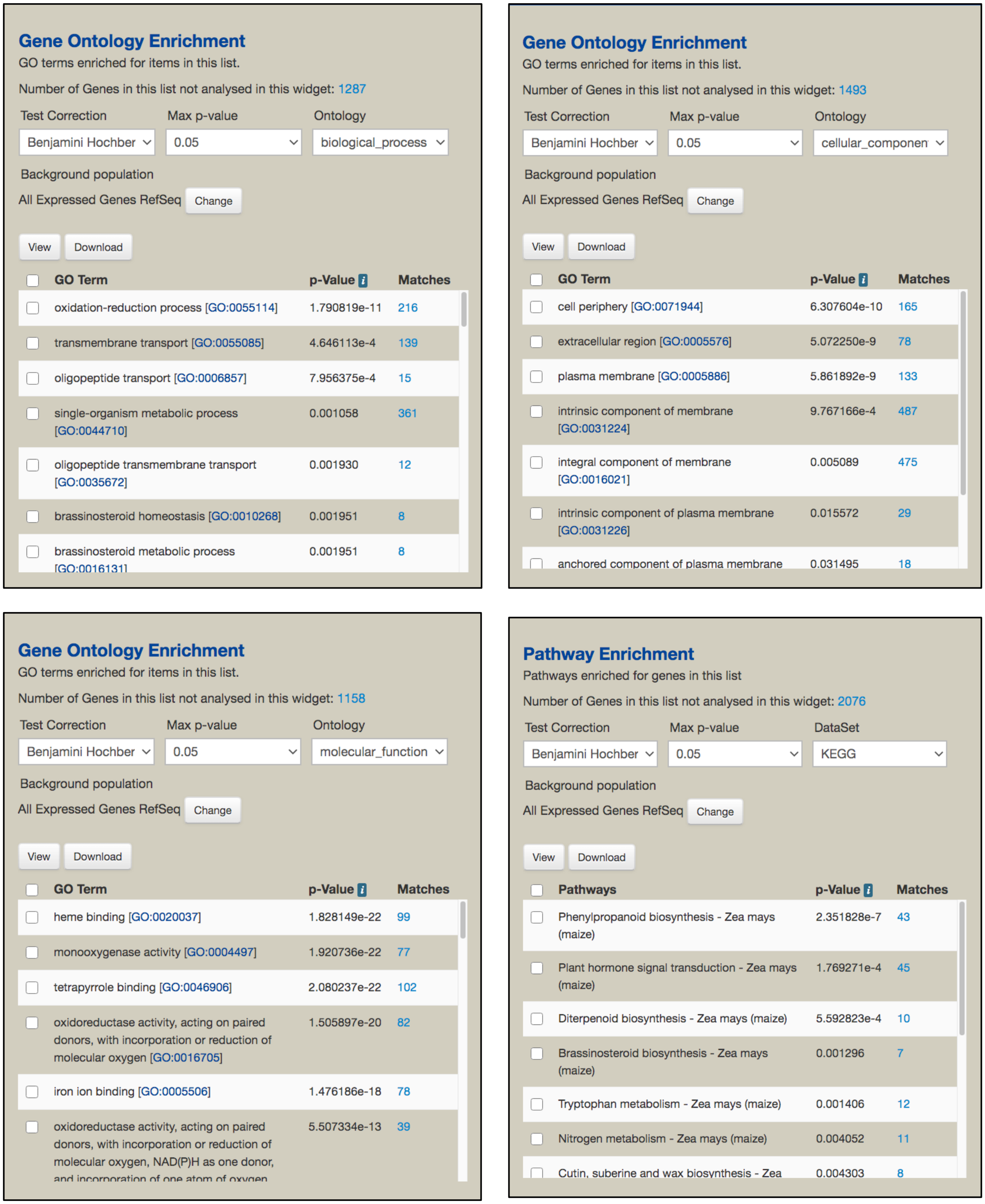

Supplementary Figure 19. Enrichment of GO and pathway terms for 'DE Genes High and Low P RefSeq' in Example 2. The background population has been changed to 'All Expressed Genes RefSeq' and test correction has been changed to 'Benjamini Hochberg'.
