# Supplementary material for: MaizeMine: A Data Mining Warehouse for the Maize Genetics and Genomics Database
Source: Front Plant Sci. 2020 Oct 22;11:592730. doi: 10.3389/fpls.2020.592730 (PMC7642280; doi:10.3389/fpls.2020.592730)
Supplement: Supplementary Data Sheet 2 — API examples. [file Data_Sheet_2.PDF]

MaizeMine API with Python Examples

InterMine client API libraries are available for Java, Perl, Python, Ruby, JavaScript, and R. Full InterMine API documentation, client libraries and additional tutorials for each of the libraries are available here: https://intermine.readthedocs.io/en/latest/web-services/#api-and-client-libraries

The MaizeMine examples in this document are performed with python2.

To get started with this tutorial, download the Python libraries using the url above or install the Python libraries via pip as follows:

```
$ pip install intermine
```

Example 1: Simple query. Supplementary Figure 20 below shows the query that will be performed in this example.

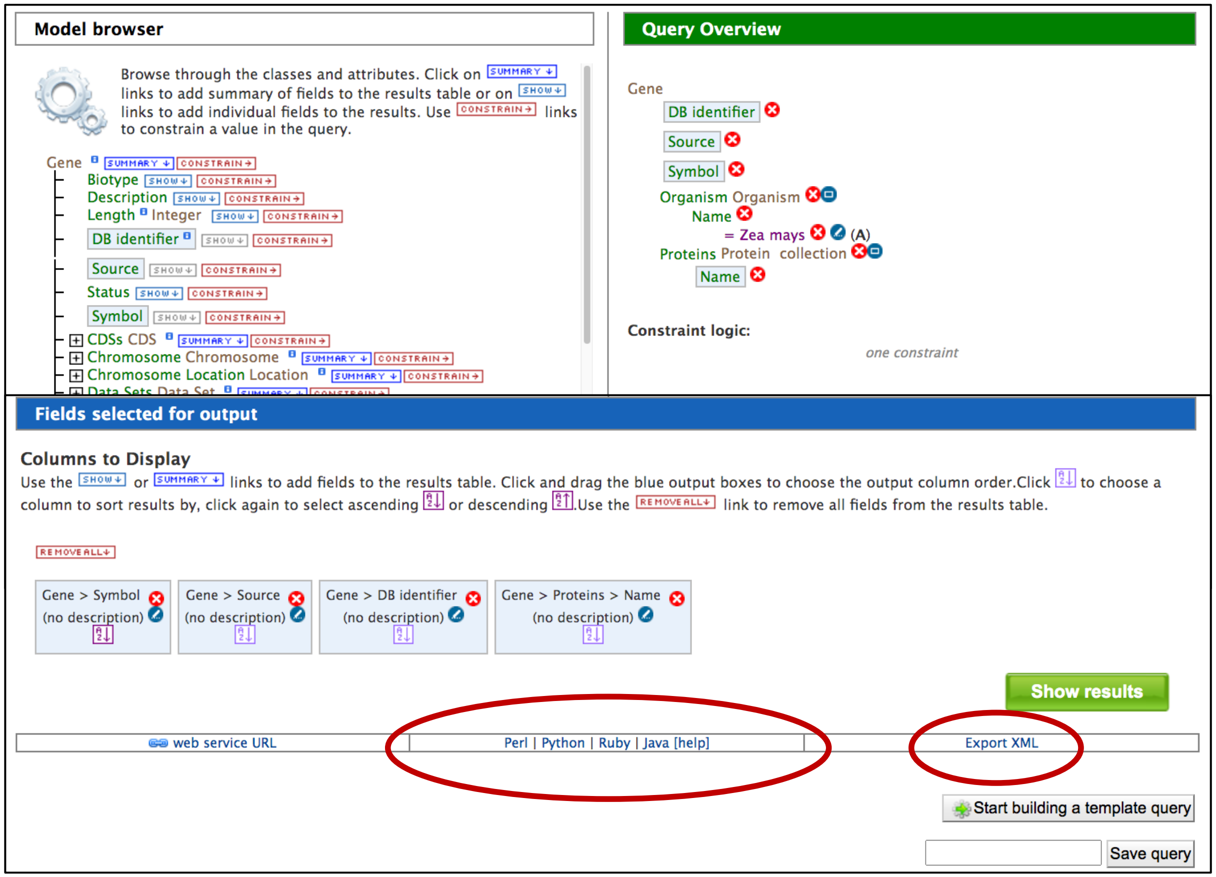

Supplementary Figure 20. The QueryBuilder page showing query to be performed in the first API example. The Query Overview shows the columns to be selected and the constraint for the Organism name "Zea mays". Clicking 'Python', in the area circled in red, provides the client Python code. Clicking 'Export XML' provides the query XML.

Clicking "Export XML" in the QueryBuilder provides the following XML:

```
<query model="genomic" view="Gene.symbol Gene.source Gene.primaryIdentifier Gene.proteins.name"
sortOrder="Gene.symbol ASC" >
  <constraint path="Gene.organism.name" op="=" value="Zea mays" code="A" />
</query>
```

Clicking 'Python' on the QueryBuilder page provides the following Python code:

```
--------------------------------------------------------
#!/usr/bin/env python

# This is an automatically generated script to run your query
# to use it you will require the intermine python client.
# To install the client, run the following command from a terminal:
#
#     sudo easy_install intermine
#
# For further documentation you can visit:
#     http://intermine.readthedocs.org/en/latest/web-services/

# The following two lines will be needed in every python script:
from intermine.webservice import Service
service = Service("http://maizemine.rnet.missouri.edu:8080/maizemine/service")

# Get a new query on the class (table) you will be querying:
query = service.new_query("Gene")

# The view specifies the output columns
query.add_view("symbol", "source", "primaryIdentifier", "proteins.name")

# Uncomment and edit the line below (the default) to select a custom sort order:
# query.add_sort_order("Gene.symbol", "ASC")

# You can edit the constraint values below
query.add_constraint("organism.name", "=", "Zea mays", code = "A")

# Uncomment and edit the code below to specify your own custom logic:
# query.set_logic("A")

for row in query.rows():
    print row["symbol"], row["source"], row["primaryIdentifier"], row["proteins.name"]
```

Sample output (first few lines):

```
LOC100278359 RefSeq 100278359 Putative uncharacterized protein
LOC100278860 RefSeq 100278860 Putative uncharacterized protein
```

```
LOC103646867 RefSeq 103646867 Alpha-humulene/(-)-(E)-beta-caryophyllene synthase
pco096967 RefSeq 100282122 (+)-neomenthol dehydrogenase
LOC100280419 RefSeq 100280419 (+)-neomenthol dehydrogenase
```

****

Example 2: Template query "Gene -> Gene Expression with Specified Threshold" (Figure 2 in the MaizeMine paper)

This example uses interactive Python via the command line. The symbols “>>>” and “…” represent the interactive Python command prompts (where “…” indicates indention). Results of the commands are shown without a command prompt.

1. Obtain the template name from the URL from the "name=" part of the url:

http://maizemine.rnet.missouri.edu:8080/maizemine/template.do?**name=gene_expression_with_constraints**&scope=global

(For this template, the name is "gene_expression_with_constraints.")

2. Get the template columns using template.views, and template.constraint_dict to get current constraints (in particular showing which ones are editable).

```
>>> from intermine.webservice import Service
>>> service = Service("http://maizemine.rnet.missouri.edu:8080/maizemine/service")
>>> template=service.get_template('gene_expression_with_constraints')
>>> template.views
['Gene.primaryIdentifier', 'Gene.expression.sample.tissueDescription',
'Gene.expression.sample.growthStage', 'Gene.expression.sample.organGroup',
'Gene.expression.sample.poName.identifier', 'Gene.expression.sample.poName.name', 'Gene.expression.FPKM',
'Gene.expression.normalizedCounts', 'Gene.expression.type']
>>> template.constraint_dict
{'A': <TemplateBinaryConstraint: Gene.primaryIdentifier = GRMZM2G094632 (editable, locked)>, u'C':
<TemplateBinaryConstraint: Gene.expression.normalizedCounts > 0 (editable, locked)>, u'B':
<TemplateBinaryConstraint: Gene.expression.FPKM > 0 (editable, locked)>}
```

For the above template, Gene.primaryIdentifier, Gene.expression.FPKM, and Gene.expression.normalizedCounts are editable.

Suppose we wish to use the template with Gene.primaryIdentifier = "GRMZM2G094632", Gene.expression.FPKM > 10, and Gene.expression.normalizedCounts > 0 as in Figure 2 in the MaizeMine paper. Run the query changing the constraints according to how they are labeled above (A, B, C). Note that the first python statement below should be entered on one line, although page constraints cause it to appear on two lines in this document. Also note that there is a tab indentation before "print(row)" on the second line.

```
>>> for row in
template.results(row="rr",A={"value":"GRMZM2G094632"},B={"op":">","value":"10"},C={"op":">","value":"0"}):
...     print(row)
...
Gene: primaryIdentifier=u'GRMZM2G094632' expression.sample.tissueDescription=u'Meotic tassel'
expression.sample.growthStage=u'V18' expression.sample.organGroup=u'Reproductive'
expression.sample.poName.identifier=u'PO:0020126' expression.sample.poName.name=u'tassel inflorescence'
expression.FPKM=7096.8735 expression.normalizedCounts=45690.6 expression.type=u'mean'
Gene: primaryIdentifier=u'GRMZM2G094632' expression.sample.tissueDescription=u'Topmost leaf'
expression.sample.growthStage=u'V3' expression.sample.organGroup=u'Leaf'
expression.sample.poName.identifier=u'PO:0009025' expression.sample.poName.name=u'vascular leaf'
expression.FPKM=20.748432 expression.normalizedCounts=128.22733 expression.type=u'mean'
Gene: primaryIdentifier=u'GRMZM2G094632' expression.sample.tissueDescription=u'Pooled leaves'
expression.sample.growthStage=u'V1' expression.sample.organGroup=u'Leaf'
expression.sample.poName.identifier=u'PO:0009025' expression.sample.poName.name=u'vascular leaf'
expression.FPKM=15.429033 expression.normalizedCounts=93.383736 expression.type=u'mean'
Gene: primaryIdentifier=u'GRMZM2G094632' expression.sample.tissueDescription=u'Whole seed'
expression.sample.growthStage=u'4 DAP' expression.sample.organGroup=u'Seed'
expression.sample.poName.identifier=u'PO:0009010' expression.sample.poName.name=u'seed'
expression.FPKM=11.154916 expression.normalizedCounts=62.035465 expression.type=u'mean'
Gene: primaryIdentifier=u'GRMZM2G094632' expression.sample.tissueDescription=u'Whole seed'
expression.sample.growthStage=u'6 DAP' expression.sample.organGroup=u'Seed'
expression.sample.poName.identifier=u'PO:0009010' expression.sample.poName.name=u'seed'
expression.FPKM=10.7042 expression.normalizedCounts=59.3552 expression.type=u'mean'
```

You can also extract one column, e,g., primaryIdentifier. Again, the first python statement should be entered on one line, and the second statement has a tab indention.

```
>>> for row in template.results(row="rr",
A={"value":"GRMZM2G094632"},B={"op":">","value":"10"},C={"op":">","value":"0"}):
...     print row["primaryIdentifier"]
...
GRMZM2G094632
GRMZM2G094632
GRMZM2G094632
GRMZM2G094632
GRMZM2G094632
```

You can save these as a Python list:

```
>>> primaryIds =list((row["primaryIdentifier"] for row in template.results(row="rr",
A={"value":"GRMZM2G094632"},B={"op":">","value":"10"},C={"op":">","value":"0"})));
>>> primaryIds
[u'GRMZM2G094632', u'GRMZM2G094632', u'GRMZM2G094632', u'GRMZM2G094632', u'GRMZM2G094632']
```

****

Example 3: List upload and using an existing list to create a new list with a constraint

To upload a list of identifiers to your MyMine account, create an array that contains the gene identifiers that would normally be pasted/uploaded using the MaizeMine List Tool. In the example below, we named the array "identifiers". On the service line, you will include your MaizeMine API token, generated in the 'Account Details' page of your MyMine account, which allows you to save the lists to your account. In the example below, the user API token is "S1C774qaB2g1C0H310Rb", but you should replace it with your own API token. The list we save in the example below is called "My Example Z. mays Genes List".

Python script code:

```
--------------------------------------------------------
#!/usr/bin/env python

# Create a list of genes from a python list of identifiers
```

frontiers

```
# The following two lines will be needed in every python script. Note the API token usage in this example.
# Replace it with your own token:
from intermine.webservice import Service
service = Service("http://maizemine.rnet.missouri.edu:8080/maizemine/service",token="S1C774qaB2g1C0H310Rb")

# Define a python list of identifiers to search on
identifiers=["Zm00001d00002","Zm00001d000017","Zm00001d000034","Zm00001d000141","Zm00001d000169","Zm00001d0
00193","Zm00001d000194","Zm00001d000195","Zm00001d000220","Zm00001d000224","AC148152.3_FG001","AC148152.3_F
G002","AC148152.3_FG008","AC149475.2_FG004"]

# Create a list from these identifiers and save it to your MaizeMine account
service.create_list(content=identifiers,list_type="Gene",name="My Example Z. mays Genes List")
---------------------------------------------------------
```

In the following code, we create a new list called “My Example Z. mays Genes List 2” that is comprised of only the genes from “My Example Z. mays Genes List” with the source “AGPv4”.

```
---------------------------------------------------------
#!/usr/bin/env python

# The following two lines will be needed in every python script. Note the API token usage in this example.
# Replace it with your own token:
from intermine.webservice import Service
service = Service("http://maizemine.rnet.missouri.edu:8080/maizemine/service",token="S1C774qaB2g1C0H310Rb")

# Get a new query on the class (table) you will be querying:
query=service.new_query("Gene")

# Add query constraints:
query.add_constraint("Gene","IN","My Example Z. mays Genes List")
query.add_constraint("source","=","AGPv4")

# Create a new list with the results of running the query on the above list, and
# save the list to your MaizeMine account:
service.create_list(query,name="My Example Z. mays Genes List 2")
---------------------------------------------------------
```

You can visit MaizeMine via the web interface to verify that these new lists have been created (Supplementary Figure S21).

****

Example 4: List upload from a file and Gene Ontology enrichment calculation on the list

In the following example a list is created by importing the identifiers from an input file named "de_gene_identifiers.txt" with one identifier per line. In this example, we used a text editor to create a file of differentially expressed genes similar to the gene list used in use-case example 2 of the MaizeMine paper (Column L of the Notes S3 tab of this file: https://nph.onlinelibrary.wiley.com/action/downloadSupplement?doi=10.1111%2Fnph.14893&file=nph14893-sup-0002-NotesS1-S5.xlsx). Again, your API token is used in the service line so that you can save the list in your MyMine account. Note that you will use your own API token, not the one shown below.

Python code to save this new list and perform enrichment for molecular function GO terms:

```
--------------------------------------------------------
#!/usr/bin/env python


# Create a list of gene ids from a text file containing identifiers and perform GO enrichment analysis

# Generate a python list of identifiers from the input file:
de_gene_list = []

# Remove the newline characters from the list
with open("de_gene_identifiers.txt") as f:
    for line in f:
        de_gene_list.append(line.rstrip())

# The following two lines will be needed in every python script. Note the API token usage in this example.
# Replace it with your own token:
from intermine.webservice import Service
service = Service("http://maizemine.rnet.missouri.edu:8080/maizemine/service",token="S1C774qaB2g1C0H310Rb")

# Create a list from these identifiers and save them to your MaizeMine account
# Also save the list objects:
l = service.create_list(content=de_gene_list,list_type="Gene",name="DE Genes API example")

# Perform gene ontology enrichment on list object l. Note that the code below should not have a newline
# until after “molecular_function")”. Also note that if the “filter=” option is not provided,
```

```
# enrichment for biological process terms will be performed:
r=l.calculate_enrichment(widget="go_enrichment_for_gene",maxp=0.05, background="AGPv3.21 All Genes",
correction="Benjamini Hochberg", filter="molecular_function")

# Display results of enrichment analysis:
for i in r:
    print(i)
----------------------------------------------------------
```

Output:

{u'matches': 130, u'p-value': 5.76703945803674e-28, u'populationAnnotationCount': 494, u'description': u'oxidoreductase activity, acting on paired donors, with incorporation or reduction of molecular oxygen', u'identifier': u'GO:0016705'}
{u'matches': 96, u'p-value': 7.363637567999759e-27, u'populationAnnotationCount': 305, u'description': u'monooxygenase activity', u'identifier': u'GO:0004497'}
{u'matches': 125, u'p-value': 6.811999565027236e-23, u'populationAnnotationCount': 525, u'description': u'heme binding', u'identifier': u'GO:0020037'}
{u'matches': 128, u'p-value': 1.4127052212553404e-21, u'populationAnnotationCount': 564, u'description': u'tetrapyrrole binding', u'identifier': u'GO:0046906'}
{u'matches': 109, u'p-value': 3.638028909957526e-20, u'populationAnnotationCount': 455, u'description': u'iron ion binding', u'identifier': u'GO:0005506'}
{u'matches': 274, u'p-value': 3.6032141025823406e-15, u'populationAnnotationCount': 1900, u'description': u'oxidoreductase activity', u'identifier': u'GO:0016491'}
{u'matches': 90, u'p-value': 2.9082889949680702e-08, u'populationAnnotationCount': 510, u'description': u'electron carrier activity', u'identifier': u'GO:0009055'}
{u'matches': 167, u'p-value': 9.566660426052002e-08, u'populationAnnotationCount': 1187, u'description': u'transporter activity', u'identifier': u'GO:0005215'}
{u'matches': 23, u'p-value': 6.221022891681447e-07, u'populationAnnotationCount': 65, u'description': u'serine-type endopeptidase inhibitor activity', u'identifier': u'GO:0004867'}
{u'matches': 24, u'p-value': 1.742534617558268e-05, u'populationAnnotationCount': 82, u'description': u'endopeptidase inhibitor activity', u'identifier': u'GO:0004866'}
{u'matches': 24, u'p-value': 1.742534617558268e-05, u'populationAnnotationCount': 82, u'description': u'peptidase inhibitor activity', u'identifier': u'GO:0030414'}
{u'matches': 24, u'p-value': 1.742534617558268e-05, u'populationAnnotationCount': 82, u'description': u'peptidase regulator activity', u'identifier': u'GO:0061134'}
{u'matches': 24, u'p-value': 1.742534617558268e-05, u'populationAnnotationCount': 82, u'description': u'endopeptidase regulator activity', u'identifier': u'GO:0061135'}
{u'matches': 34, u'p-value': 0.00020672921858776072, u'populationAnnotationCount': 162, u'description': u'dioxygenase activity', u'identifier': u'GO:0051213'}

{u'matches': 1021, u'p-value': 0.0002151508566172066, u'populationAnnotationCount': 10567, u'description': u'catalytic activity', u'identifier': u'GO:0003824'}
{u'matches': 27, u'p-value': 0.0003905591830185789, u'populationAnnotationCount': 118, u'description': u'oxidoreductase activity, acting on paired donors, with incorporation or reduction of molecular oxygen, 2-oxoglutarate as one donor, and incorporation of one atom each of oxygen into both donors', u'identifier': u'GO:0016706'}
{u'matches': 116, u'p-value': 0.0005645748318024692, u'populationAnnotationCount': 877, u'description': u'transmembrane transporter activity', u'identifier': u'GO:0022857'}
{u'matches': 116, u'p-value': 0.0022765582718545212, u'populationAnnotationCount': 905, u'description': u'nucleic acid binding transcription factor activity', u'identifier': u'GO:0001071'}
{u'matches': 116, u'p-value': 0.0022765582718545212, u'populationAnnotationCount': 905, u'description': u'transcription factor activity, sequence-specific DNA binding', u'identifier': u'GO:0003700'}
{u'matches': 36, u'p-value': 0.00310762017296529, u'populationAnnotationCount': 201, u'description': u'peroxidase activity', u'identifier': u'GO:0004601'}
{u'matches': 36, u'p-value': 0.004100500548455624, u'populationAnnotationCount': 204, u'description': u'oxidoreductase activity, acting on peroxide as acceptor', u'identifier': u'GO:0016684'}
{u'matches': 80, u'p-value': 0.005709928421767387, u'populationAnnotationCount': 590, u'description': u'transferase activity, transferring hexosyl groups', u'identifier': u'GO:0016758'}
{u'matches': 6, u'p-value': 0.0065952512994922954, u'populationAnnotationCount': 10, u'description': u'cis-zeatin O-beta-D-glucosyltransferase activity', u'identifier': u'GO:0050502'}
{u'matches': 401, u'p-value': 0.04052470407731687, u'populationAnnotationCount': 3954, u'description': u'cation binding', u'identifier': u'GO:0043169'}
{u'matches': 399, u'p-value': 0.042662920952474925, u'populationAnnotationCount': 3937, u'description': u'metal ion binding', u'identifier': u'GO:0046872'}

Again, you can visit MaizeMine via the web interface to verify that this new list has been created (Supplementary Figure S21).

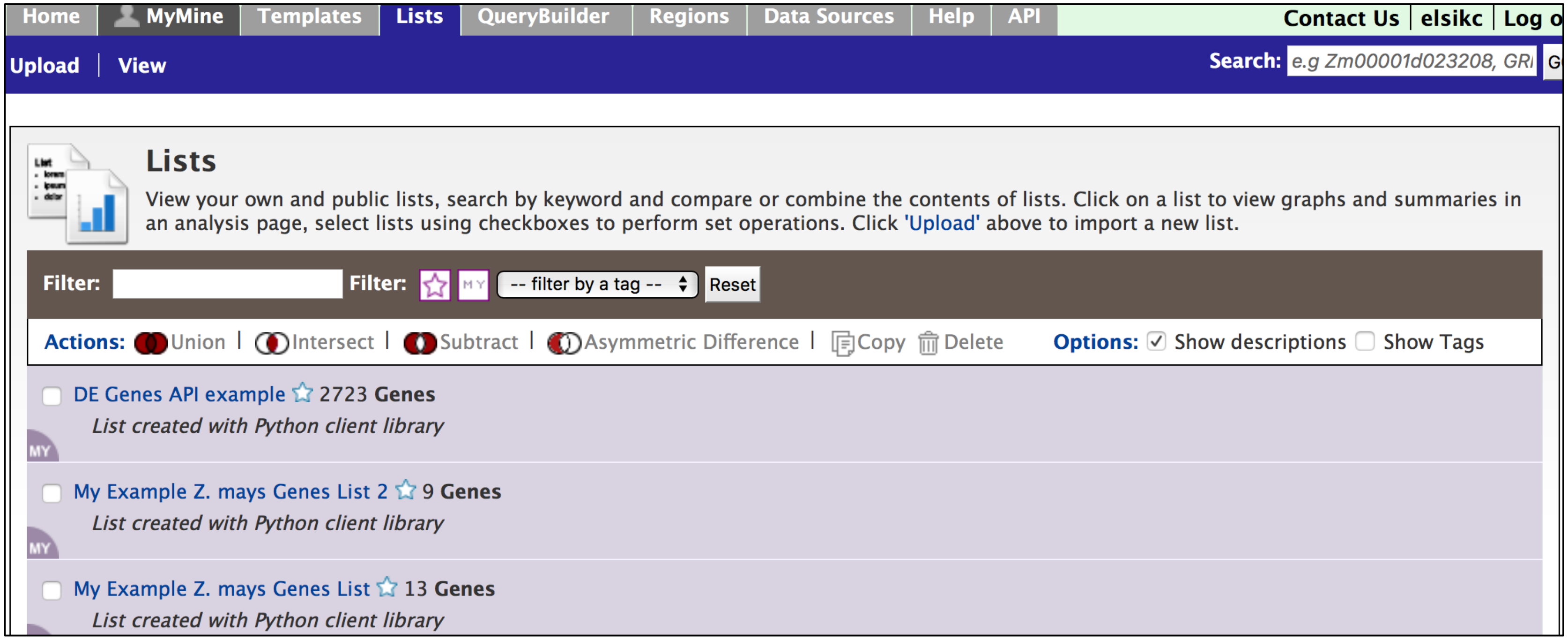

Supplementary Figure 21. The List View page verifying that the lists created in Examples 3 and 4 have been saved in the user account.
